# Human-associated NDM-5-producing multidrug-resistant *Escherichia coli* detected in retail beef and pork in Hungary, 2021

**DOI:** 10.3389/fbinf.2026.1793862

**Published:** 2026-03-24

**Authors:** Mirena Ivanova, Joana Mourão, Judit Szarvas, Elif S. Tosun, Niamh Lacy-Roberts, Natasia Rebekka Thornval, Zita Záborcki, Szilárd Jánosi, Raquel Garcia-Fierro, Pierre-Alexandre Beloeil, Ernesto Liebana, Beatriz Guerra, Rene S. Hendriksen, Jette S. Kjeldgaard

**Affiliations:** 1 European Union Reference Laboratory for Antimicrobial Resistance (EURL-AR), Research Group for Global Capacity Building, Technical University of Denmark, Kongens Lyngby, Denmark; 2 Research Group for Genomic Epidemiology, National Food Institute, Technical University of Denmark, Kongens Lyngby, Denmark; 3 The Novo Nordisk Foundation Center for Basic Metabolic Research, Faculty of Health and Medical Sciences, University of Copenhagen, Copenhagen, Denmark; 4 National Food Chain Safety Office, Veterinary Diagnostic Directorate, Budapest, Hungary; 5 European Food Safety Authority, Parma, Italy

**Keywords:** blaNDM-5, carbapenemase-producing Enterobacterales, *Escherichia coli* ST405, horizontal gene transfer, IncFII-IncFIB plasmid, multidrug resistance, one health surveillance

## Abstract

**Background:**

Carbapenem-resistant Enterobacterales pose a significant public health threat, particularly when detected in food-producing animals and retail meat. Although carbapenems are not used in European Union animal production, sporadic cases of carbapenemase-producing *Escherichia coli* have emerged across multiple European countries since 2019. The detection of human-associated carbapenemase genes in meat raises concerns about potential transmission to humans through the food chain.

**Methods:**

In this study, we characterize three multidrug-resistant (MDR) *E. coli* isolates harboring *bla*_NDM-5_ recovered from retail beef and pork in Hungary in 2021. *E. coli* isolates were subjected to phenotypic antimicrobial susceptibility testing using broth microdilution, conjugation experiments, and genotypic characterization through whole-genome sequencing using Illumina and Oxford Nanopore platforms. Hybrid assemblies enabled comprehensive comparative genomic and plasmid analyses.

**Results:**

All three isolates belonged to the human-associated uropathogenic clone ST405 (O102:H6) and were clonally related with a maximum of two single nucleotide polymorphisms. They exhibited identical genomic profiles conferring resistance to carbapenems, cephalosporins, fluoroquinolones, tetracycline, and azithromycin. Comparative genomic analysis revealed close genetic relationships with human clinical isolates from Australia and the United Kingdom, suggesting international dissemination. The *bla*_NDM-5_ gene was located on conjugative IncFII-IncFIB hybrid plasmids (approximately 132 kb) closely related to clinical plasmids from human isolates in the United States, differing only by the absence of a *bla*_CTX-M-15_-ISEcp1 transposition unit.

**Conclusion:**

The detection of human-associated *bla*_NDM-5_-carrying *E. coli* ST405 in retail meat represents a serious food safety concern, highlighting potential transmission routes to humans and emphasizing the need for enhanced surveillance and epidemiological investigations.

## 1 Introduction

Carbapenems are last-resort antibiotics for treating serious infections caused by multidrug-resistant (MDR) Gram-negative bacteria, and the emergence of carbapenemase-producing Enterobacterales (CPE) constitutes a major global public health concern due to severely limited therapeutic options and increased morbidity and mortality (Bonomo et al., 2018). Although carbapenems are not approved for use in food-producing animals in the European Union (EU), CPE have increasingly been detected in livestock and retail meat, indicating that non-human reservoirs may contribute to the dissemination of carbapenem resistance within a One Health framework (Carfora et al., 2022; Hendriksen et al., 2023). Harmonized European surveillance has documented an escalating occurrence of CPE in food-producing animals and meat, with sporadic detections in 2020–2021 (European Food Safety Authority (EFSA) and European Centre for Disease Prevention and Control, 2023) progressing to more frequent reports from multiple Member States, including Italy, Germany, Spain, Czechia, and Hungary, by 2022–2023 (European Food Safety Authority and European Centre for Disease Prevention and Control, 2025). In these monitoring programs, *bla*_OXA-181_ has been the most frequently detected carbapenemase gene in food-producing animals, followed by *bla*_NDM-5_, *bla*_OXA-48_, and *bla*_VIM-1_ (ECDC, 2023; European Food Safety Authority and European Centre for Disease Prevention and Control, 2025), underscoring the growing diversity of carbapenemase determinants in the animal sector. Among these enzymes, New Delhi metallo-β-lactamase NDM-5 has emerged as a particularly prevalent variant, with a marked increase in *Escherichia coli* isolates across the EU/EU/European Economic Area (EEA) from 2012 to 2022, and sequence type (ST) 405 identified as one of the major epidemic clones associated with this carbapenemase (European Centre for Disease Prevention and Control, 2023; Linkevicius et al., 2023; Xia et al., 2024).

*E*. *coli* ST405 is a clinically significant extraintestinal pathogenic lineage linked to uropathogenic and other invasive infections, frequently associated with high patient mortality and MDR phenotypes (Chowdhury et al., 2019; Hans et al., 2023; Mujahid et al., 2024). This high-risk clone has been reported worldwide and is increasingly connected to carbapenem resistance, often co-harboring *bla*_NDM-5_ and extended-spectrum β-lactamase genes such as *bla*_CTX-M-15_ on conjugative plasmids, a combination that exacerbates treatment challenges and facilitates persistence in both community and healthcare settings (Mujahid et al., 2024). Recent genomic studies of MDR *E. coli* isolated from human urinary tract infections have highlighted the prominent role of ST405 and related high-risk lineages in the global dissemination of carbapenem resistance and ESBL determinants, reinforcing concerns about their potential spread beyond clinical environments (Mujahid et al., 2024; Sivarajan et al., 2025). This ST has also been associated with enhanced virulence traits and severe clinical outcomes, representing a concerning convergence of antimicrobial resistance and pathogenic potential (Chowdhury et al., 2019; Peri et al., 2020; Mujahid et al., 2024). The detection of human-associated CPE clones such as ST405 in the food chain therefore raises important questions regarding transmission dynamics, contamination sources, and the extent to which animal and food reservoirs contribute to the circulation of these high-risk lineages (Sivarajan et al., 2025).

The dissemination of carbapenem resistance is primarily driven by horizontal gene transfer mediated by mobile genetic elements, particularly conjugative plasmids. IncF plasmids, including IncFII and IncFIB replicon types, represent some of the most prevalent vectors for *bla*_NDM-5_ and *bla*_CTX-M-15_ dissemination in *E. coli* worldwide (Zhang et al., 2021; Turton et al., 2022; Mujahid et al., 2024; Xia et al., 2024), promoting the spread of carbapenem and extended-spectrum β-lactam resistance across diverse ecological niches. These plasmids typically display mosaic backbones with conserved core regions and variable accessory modules. This architecture facilitates the accumulation, recombination, and co-selection of multiple resistance determinants through insertion sequence-mediated events (Chowdhury et al., 2019; Zhang et al., 2021; Turton et al., 2022). Their efficient conjugative transfer enables the movement of *bla*_NDM-5_-bearing plasmids between different *E. coli* lineages and hosts, including between humans and animals, and shared plasmid backbones have been documented among isolates from clinical, animal, and environmental sources. Consequently, the detection of CPE in food-producing animals and retail meat, combined with evidence of plasmid-mediated exchange between human and animal isolates, raises critical One Health concerns regarding the potential for bidirectional transmission along the human–animal–food interface (Chakraborty et al., 2021; Zhang et al., 2021; Sivarajan et al., 2025).

Epidemiological linkages between human and animal CPE isolates have been increasingly suggested (Carfora et al., 2022; Fu et al., 2024), with evidence pointing to possible bidirectional transmission events that complicate attribution of sources. Harmonized antimicrobial resistance monitoring in the EU primarily targets commensal indicator *E. coli*, yet the identification of human-associated high-risk clones in retail meat suggests potential contamination from human reservoirs, as illustrated by investigations in Italy where *bla*_OXA-48_-harboring *E. coli* isolates in meat were traced back to human sources (Carfora et al., 2022). At the same time, comprehensive source-tracing studies that connect CPE detected in retail meat to specific animal, environmental, or human reservoirs remain limited, leaving substantial knowledge gaps regarding the directionality and drivers of transmission. The European Centre for Disease Prevention and Control (ECDC) has also reported a deteriorating epidemiological situation for CPE in the EU/EEA, including increasing detection of high-risk *E. coli* lineages carrying carbapenemase genes such as *bla*_NDM-5_, *bla*_KPC_, and *bla*_OXA-48_-like variants, which pose risks for both hospital- and community-associated transmission (European Centre for Disease Prevention and Control, 2025). Addressing these gaps requires integrated genomic and epidemiological investigations that jointly analyze isolates from human, animal, food, and environmental compartments.

In this study, three clonally-related multidrug-resistant *E. coli* ST405 isolates carrying *bla*_NDM-5_ and *bla*_CTX-M-15_ on conjugative IncFII–IncFIB hybrid plasmids were recovered from retail beef and pork in Hungary in 2021. Using whole-genome sequencing (WGS), comparative genomics, and conjugation experiments, this work provides a comprehensive genomic, epidemiological, and phenotypic characterization of these isolates, examines their genetic relationship to international human clinical *E. coli* ST405 strains, and evaluates the horizontal transferability of their resistance plasmids. By situating these findings within a One Health framework, the study aims to improve understanding of the role of retail meat in the dissemination of NDM-5-producing high-risk *E. coli* lineages and to elucidate the potential public health implications of their presence in the food chain.

## 2 Materials and methods

### 2.1 Bacterial isolates and antimicrobial susceptibility testing

The 3 *E. coli* isolates were collected through specific monitoring of ESBL-/AmpC-/CP-producing *E. coli* in 2021 using methodology recommended by the European Union Reference Laboratory for Antimicrobial Resistance (EURL-AR) for Feed, Food and Animal Health (Hendriksen et al., 2023). As part of the EURL-AR confirmatory testing exercise, the isolates recovered from retail beef and pork were characterized by broth microdilution method for determination of Minimum Inhibitory Concentration (MIC) using EUVSEC2 and EUVSEC3 Sensititre™ panels (Thermo Fisher Scientific, MA, United States) following the manufacturer’s instructions. The MIC values were interpreted according to EUCAST epidemiological cut-off values (http://eucast.org/) or EFSA-defined surveillance Epidemiological cut-off values (ECOFFs) (European Food Safety Authority et al., 2021).

### 2.2 Whole genome sequencing

For short-read Illumina WGS, total DNA was extracted using the DNeasy Blood and Tissue Kit (Cat. No. 69504, Qiagen, Germany) and quantified using the Qubit 4 Fluorometer (Cat. No. Q33238, Invitrogen) according to manufacturer’s instructions. Sequencing libraries were constructed using the Nextera XT Library Prep Kit (Cat. No. FC-131-1096, Illumina, San Diego, CA, United States), loaded on a NextSeq 500/550 Mid Output v2.5 Kit (300 cycles) flowcell, and pair-end sequenced on a NextSeq 500 Illumina platform.

For long-read Oxford Nanopore Technologies (ONT) sequencing, total DNA was extracted using the Quick-DNA HMW Magbead Kit (Cat. No. D6060, Zymo Research) following the manufacturer’s protocol with minor modifications. Due to technical difficulties during total DNA extraction and sequencing, plasmid DNA from isolate M2021_10043982_E was alternatively extracted using the QIAGEN Plasmid Midi kit (Cat. No. 12181, QIAGEN, Germany) as described in Ivanova et al. (2024) (Ivanova et al., 2024). Therefore, a complete closed genome assembly could not be obtained for this isolate, though chromosomal characterization was performed using Illumina short-read data. DNA concentration was measured using the Qubit 4 Fluorometer and stored at 4 °C until library preparation. One μg high-quality DNA was used for library preparation with the Ligation gDNA Native Barcoding Kit 24 V14 (SQK-NBD114.24, Oxford Nanopore Technologies, Oxford) following the manufacturer’s instructions with modifications mainly involving increased incubation times and loaded onto a R10.4 flow cell. The flow cell was sequenced on the GridION platform for 72 h and basecalling was performed live in MinKNOW with super-accurate (SUP) basecalling and sequencing reads with Phred score <9 and length <200 bp were filtered out by MinKNOW.

### 2.3 Data analysis

Illumina raw sequencing reads were quality-checked using FastQC v0.11.9 (Andrews, 2010) and trimmed using bbduk2 (part of the suite bbtools v36.49) (Bushnell et al., 2017). The quality of the long Oxford Nanopore (ONT) reads were evaluated with NanoPlot v1.33.0 (De Coster and Rademakers, 2023) and trimmed to a minimum read length of 5 kbp (--min_length 5000) by Filtlong v0.2.0 (https://github.com/rrwick/Filtlong) (details in Supplementary Figure S1 **)**. High-quality long ONT reads were *de novo* assembled using Flye v2.9 (Kolmogorov et al., 2019) and an estimated genome size of 5 Mbp, and subsequently corrected with one of the trimmed Illumina short reads with Polypolish v0.5.0 (Wick and Holt, 2022) using default settings. Full annotation was performed using Bakta v1.11.4 (Schwengers et al., 2021), and Insertion Sequences (IS) were predicted using ISFinder (Siguier, 2006). Assembly statistics, including number of contigs, N50, GC content, were generated using Quast v5.2.0 (Gurevich et al., 2013) (Supplementary Table S1).

Antimicrobial resistance (AMR) genes were identified using ABRicate v1.0.1 (https://github.com/tseemann/abricate) and the ResFinder v4.7.2 database (version 2024-03-22) (Bortolaia et al., 2020) and the associated chromosomal mutations were searched using PointFinder.py (https://bitbucket.org/genomicepidemiology/pointfinder/src/master/) and the PointFinder database (version 2024-03-08) (Zankari et al., 2017). Virulence factors were detected using the Virulence Factor Database (VFDB) (Liu et al., 2022) and Ecoli_vf in ABRicate. Seven-gene Multilocus Sequence Typing (MLST) was determined using mlst (https://github.com/tseemann/mlst). Serotype determination was conducted using SerotypeFinder (Joensen et al., 2015). Plasmid replicon characterization and typing were performed by PlasmidFinder in ABRicate and pMLST (Carattoli et al., 2014), respectively. A minimum identity and coverage threshold of 90% was applied for all gene searches.

### 2.4 Comparative genomic and phylogenetic analysis

The clonal relatedness of the three Hungarian *E. coli* isolates was assessed by single nucleotide polymorphism (SNP)-based analysis using CSI Phylogeny (Kaas et al., 2014) with default parameters on the trimmed Illumina fastq reads. Core-genome MLST (cgMLST) scheme and hierarchical clustering of cgMLST (HierCC) within Enterobase (https://enterobase.warwick.ac.uk/) were further used to validate the results. To compare the three Hungarian ST405 genomes with globally available sequences, Enterobase was queried for the most similar genomes based on the same HierCC group (HC2). SNP-based analysis between the Hungarian *E. coli* isolates and the most similar *E. coli* genomes in Enterobase was then conducted using CSI Phylogeny with default parameters on the trimmed Illumina fastq reads as described above. High-quality SNPs in core regions shared by all genomes were aligned and the resulting core-SNP alignment used to infer a maximum-likelihood (ML) phylogeny with IQ-TREE2 v2.1.3 (Minh et al., 2020) with the GTR + G nucleotide substitution model and 1000 ultrafast bootstrap replicates (--ufboot 1000). The tree was visualised and annotated in iTOL (Letunic and Bork, 2019).

The *bla*_NDM-5_-harbouring IncFII-IncFIB plasmid sequences of the 3 *E. coli* isolates were subjected to BLAST search in the National Center for Biotechnology Information (NCBI) core nucleotide database (accessed September 2023). Sequences with varying coverage values to the queries were selected to perform comparative genomic analysis using BLAST Ring Image Generator (BRIG) (Alikhan et al., 2011). Pygenomeviz was utilized for linear comparison of the MDR regions of the plasmids (https://moshi4.github.io/pyGenomeViz/).

### 2.5 Conjugation experiments

Conjugation experiments were performed as previously described (Element et al., 2023). Donor *E. coli* M2021_10044802_2_E and recipient rifampicin resistant *E. coli* K12 20R764 strains were grown overnight in Luria broth (LB) supplemented with meropenem (8 mg/L) and rifampicin (50 mg/L), respectively. The donor and recipient strains were initially mixed at a 1:10 ratio and then diluted 1:5 in LB for static incubation at 37 °C for 20 h. Putative transconjugants (TCs) were selected on MacConkey agar supplemented with 2 mg/L meropenem and 50 mg/L rifampicin and colonies were sequenced by Illumina. DNA extraction, library preparation and bioinformatic analysis of the TCs were performed as described for the donor strains. Additionally, broth microdilution assays were carried out to determine the antimicrobial susceptibility profile of the recipient and the TCs as described above. Phenotypic and genomic data of the TCs were compared to those of the donor and recipient isolates.

## 3 Results

The three isolates exhibited identical antibiotic resistance profiles, demonstrating concordant resistance phenotypes and genetic determinants to the following antimicrobials and genes: ampicillin (*bla*_TEM-1_), cephalosporins (ceftazidime, cefotaxime, cefoxitin and cefepime) (*bla*_CTX-M-15_), carbapenems (meropenem, ertapenem and imipenem) (*bla*_NDM-5_), as well as sulfamethoxazole (*sul1*), trimethoprim (*dfrA12*), ciprofloxacin and nalidixic acid (*qepA4* and point mutations in *gyrA*, *parC*, and *parE* genes), tetracycline (*tet*(B), and azithromycin (full *mph*(A) operon) (Table 1; Supplementary Table S2). All isolates belonged to ST405 and serotype O102:H6. The ST405-O102:H6 combination identified in the three Hungarian isolates is consistent with global epidemiological patterns of carbapenem-resistant *E. coli*, as O102:H6 is among the top five most frequent serotypes (4.8%) *bla*_NDM_-producing *E. coli* worldwide, and ST405 represents an internationally disseminated high-risk clone associated with multidrug resistance (Xia et al., 2024).

**TABLE 1:** Antimicrobial resistance profiles and associated genetic determinants of the three *bla*_NDM-5_-producing *E. coli* ST405 isolates and transconjugant TC_M2021-10044802/2-E. Complete antimicrobial resistance profiles are given in Supplementary Table S2.

	D_M2021-10044802/2-E			TC_M2021-10044802/2-E^a^			M2021-10043982-E			M2021-10044824/1-E		
Antibiotic	AMR phenotype^b^	MIC (mg/L)	Genotype	AMR phenotype^b^	MIC (mg/L)	Genotype	AMR phenotype^b^	MIC (mg/L)	Genotype	AMR phenotype^b^	MIC (mg/L)	Genotype
Ampicillin	R	>32	*bla*_TEM-1B,_ *bla*_CTX-M-15,_ *bla*_NDM-5_	R	>32	*bla*_TEM-1B,_ *bla*_CTX-M-15,_ *bla*_NDM-5_	R	>32	*bla*_TEM-1B,_ *bla*_CTX-M-15,_ *bla*_NDM-5_	R	>32	*bla*_TEM-1B,_ *bla*_CTX-M-15,_ *bla*_NDM-5_
Azithromycin	R	64	*mph*(A)	R	32	*mph*(A)	R	64	*mph*(A)	R	64	*mph*(A)
Cefepime	R	>32	*bla*_CTX-M-15,_ *bla*_NDM-5_	R	16	*bla*_CTX-M-15,_ *bla*_NDM-5_	R	>32	*bla*_CTX-M-15,_ *bla*_NDM-5_	R	>32	*bla*_CTX-M-15,_ *bla*_NDM-5_
Cefotaxime	R	>4	*bla*_CTX-M-15,_ *bla*_NDM-5_	R	>4	*bla*_CTX-M-15,_ *bla*_NDM-5_	R	>4	*bla*_CTX-M-15,_ *bla*_NDM-5_	R	>4	*bla*_CTX-M-15,_ *bla*_NDM-5_
Cefotaxime/Clavulanic acid	R	>64/4	*bla*_CTX-M-15,_ *bla*_NDM-5_	R	>64/4	*bla*_CTX-M-15,_ *bla*_NDM-5_	R	>64	*bla*_NDM-5_	R	>64	*bla*_CTX-M-15,_ *bla*_NDM-5_
Ceftazidime	R	>8	*bla*_CTX-M-15,_ *bla*_NDM-5_	R	>8	*bla*_CTX-M-15,_ *bla*_NDM-5_	R	>8	*bla*_CTX-M-15,_ *bla*_NDM-5_	R	>8	*bla*_CTX-M-15,_ *bla*_NDM-5_
Ceftazidime/Clavulanic acid	R	>128/4	*bla*_CTX-M-15,_ *bla*_NDM-5_	R	>128/4	*bla*_CTX-M-15,_ *bla*_NDM-5_		>128	*bla*_NDM-5_	R	>128	*bla*_CTX-M-15,_ *bla*_NDM-5_
Ciprofloxacin	R	>8	*qepA, gyrA* (p.S83L), *gyrA* (p.D87N), *parE* (p.S458A), *parC* (p.S80I)	R	0.12	*qepA*	R	>8	*qepA, gyrA* (p.S83L), *gyrA* (p.D87N), *parE* (p.S458A), *parC* (p.S80I)	R	>8	*qepA, gyrA* (p.S83L), *gyrA* (p.D87N), *parE* (p.S458A), *parC* (p.S80I)
Cefoxitin	R	>64	*bla*_NDM-5_	R	>64	*bla*_NDM-5_	R	>64	*bla*_NDM-5_	R	>64	*bla*_NDM-5_
Ertapenem	R	>2	*bla*_NDM-5_	R	>2	*bla*_NDM-5_	R	>2	*bla*_NDM-5_	R	>2	*bla*_NDM-5_
Imipenem	R	8	*bla*_NDM-5_	R	2	*bla*_NDM-5_	R	8	*bla*_NDM-5_	R	16	*bla*_NDM-5_
Meropenem	R	16	*bla*_NDM-5_	R	2	*bla*_NDM-5_	R	16	*bl*a_NDM-5_	R	16	*bla*_NDM-5_
Nalidixic acid	R	>64	*gyrA* (p.S83L), *gyrA* (p.D87N), *parE* (p.S458A), *parC* (p.S80I)	S	≤4	-	R	>64	*gyrA* (p.S83L), *gyrA* (p.D87N), *parE* (p.S458A), *parC* (p.S80I)	R	>64	*gyrA* (p.S83L), *gyrA* (p.D87N), *parE* (p.S458A), *parC* (p.S80I)
Sulfamethoxazole	R	512	*sul1* (99.76% coverage)	S^c^	≤8	*sul1*	R	>512	*sul1*	R	512	*sul1*
Tetracycline	R	>32	*tet*(B)	R	>32	*tet*(B)	R	>32	*tet*(B)	R	>32	*tet*(B)
Trimethoprim	R	16	*dfrA12*	R	>16	*dfrA12*	R	16	*dfrA12*	R	>16	*dfrA12*
Temocillin	R	>128	*bla*_NDM-5_	R	32	*bla*_NDM-5_	R	>128	*bla*_NDM-5_	R	128	*bla*_NDM-5_

-: No AMR, genes or chromosomal mutations detected.

^a^ The antimicrobial susceptibility profiles were tested by the broth microdilution assay using EUVSEC2 and EUVSEC3 Sensititre™ panels (Thermo Fisher Scientific, MA, United States of America) according to the manufacturer’s instructions. The obtained MIC values were interpreted according to EUCAST epidemiological cut-off values (http://eucast.org/) or EFSA-defined surveillance ECOFFs (European Food Safety Authority et al., 2021).

^b^ The recipient strain *E. coli* K12 20R764 (ST10, rifampicin-resistant) exhibited susceptibility to all antimicrobials tested in this study, with MIC, values of ≤0.015 mg/L for ertapenem; ≤0.03 mg/L for ciprofloxacin and meropenem; ≤0.06 mg/L for cefepime; ≤0.12 mg/L for imipenem; ≤0.25 mg/L for cefotaxime, ceftazidime, trimethoprim, and tigecycline; ≤0.5 mg/L for gentamicin; ≤1 mg/L for colistin; ≤2 mg/L for tetracycline; ≤4 mg/L for amikacin, ampicillin, azithromycin, cefoxitin, and nalidixic acid; ≤8 mg/L for sulfamethoxazole and chloramphenicol; 8 mg/L for temocillin; ≤0.06/4 mg/L for cefotaxime/clavulanic acid; and ≤0.12/4 mg/L for ceftazidime/clavulanic acid. Bold typeface denotes antimicrobial resistance phenotypes in TCs, that were successfully transferred from and correspond to those of the donor strain.

^c^ Despite carrying *sul1*, the TC, repeatedly showed a MIC ≤8 mg/L.

The three isolates harbored an extensive repertoire of virulence factors characteristic of extraintestinal pathogenic *E. coli* (ExPEC), including multiple iron acquisition systems (yersiniabactin *ybt*, enterobactin *ent*/*fep* and heme uptake *chuA*–*chuS* clusters), three fimbrial adhesin types (type 1 fimbriae *fim* genes, P fimbriae *pap* genes and the *ecp* operon for *E. coli* common pilus), capsule biosynthesis genes (*kps* cluster), Type III secretion system (T3SS) effectors, curli fiber components (*csg* operon), and additional surface-associated proteins (Supplementary Table S3). Comparative profiling revealed a highly conserved core virulome, with 72 of 76 VFDB virulence genes (94.7%) shared by all three isolates. The four variable genes were confined to the T3SS effector repertoire, with *espX1* absent only from M2021_10044824_1_E, *espY3* absent only from M2021_10044802_2_E and *espX4* and *espY4* uniquely detected in M2021_10043982_E. The iron acquisition arsenal was particularly comprehensive, comprising 32 genes across the yersiniabactin (11 genes), enterobactin (13 genes), and heme uptake (8 genes) systems, and was complemented by diverse fimbrial adhesins together with intimin-like adhesin *fdeC* and outer membrane protein A (*ompA*), underscoring the multifactorial adherence and nutrient acquisition potential of these ST405 strains. Supplementary analysis using an alternative virulence gene database (Ecoli-vf) from ABRicate additionally identified *eaeX*, an invasiveness-associated gene previously reported as a distinctive marker of ST405 among carbapenem-resistant *E. coli*, in all three isolates.

**FIGURE 1:**
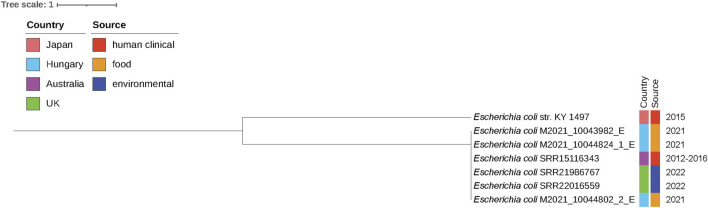
Maximum-likelihood phylogenetic tree showing single-nucleotide polymorphisms (SNPs) difference between the three Hungarian *E. coli* isolates and other closely related *E. coli* ST405 isolates, identified in Enterobase. The three Hungarian isolates had between 0 and 2 SNPs difference to 2 *E. coli* isolates recovered from environmental sources in the United Kingdom and a human isolate from Australia (Sherry et al., 2019). These isolates had also identical plasmid and AMR gene content as the three Hungarian strains. Isolate *E. coli* KY1497 ST405 isolated from human urine in Japan, also harboring the IncFIB-IncFII plasmid, was used as an outgroup and to root the tree. The difference between *E. coli* KY1497 and the other isolates in the phylogeny is 2,118–2,153 SNPs. Due to the small number of core SNPs among the isolates (0–2 SNPs), except for isolate *E. coli* KY1497, most internal branches lack meaningful bootstrap support, therefore, they are not shown.

Comparative genomic analysis confirmed that the 3 *E. coli* ST405 isolates were clonally related, despite being recovered from the meat of different animal species. Single nucleotide polymorphism (SNP)-based analysis revealed a maximum of two SNPs within their shared genomic regions. This similarity was further confirmed using the core-genome MLST (cgMLST) scheme and hierarchical clustering of cgMLST (HierCC) within Enterobase, with all strains belonging to the same HC2-172694 group. Upon comparing the three Hungarian ST405 genomes with the most similar ones available in Enterobase, a close genetic relationship (HC2-172694) was observed with three genomes sharing the same ST and AMR profiles (including *bla*_NDM-5_), one from a human clinical sample in Australia (sample accession number SAMN20033204) (Sherry et al., 2019) and two of unknown origin from the United Kingdom (SAMN31390547, SAMN31419961) (unpublished data). Maximum likelihood phylogeny generated from the core-SNP alignment of the strains from Hungary, the United Kingdom and Australia differed by only 0-2 SNPs, even though they originated from different sources (Figure 1).

**FIGURE 2:**
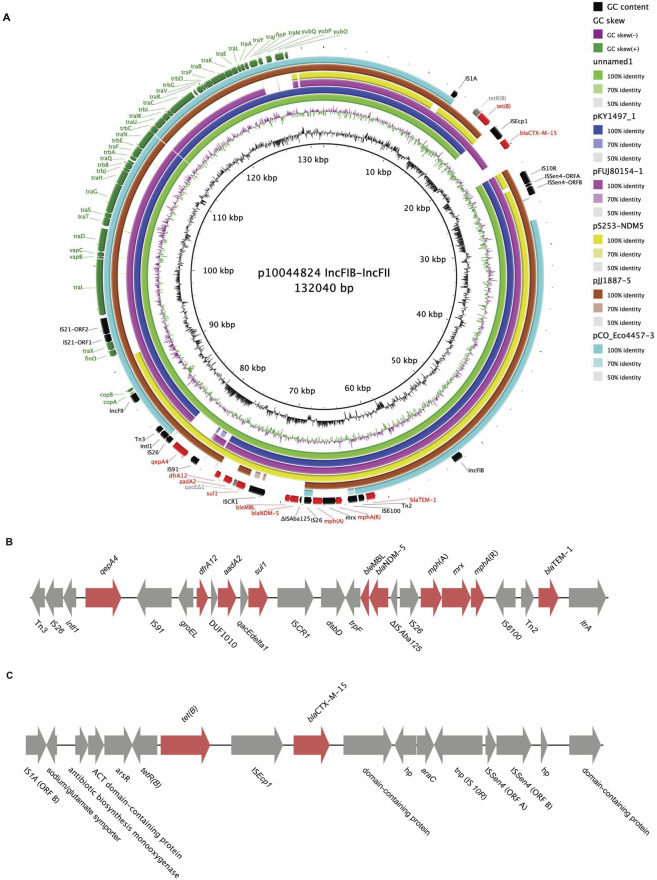
**(A)** BLAST atlas diagram of plasmid p10044824_1 of *E. coli* strain M2021_10044824_1_E with other IncFIB-IncFII plasmid complete sequences retrieved from NCBI. From inner to outer ring: *E. coli* strain AR_452 plasmid unnamed1 (unpublished), *E. coli* KY1497 plasmid pKY1497_1 (Takayama et al., 2020), *E. coli* FUJ80154 plasmid pFUJ80154-1 (unpublished), *E. coli* strain Survcare253 plasmid pS253-NDM5 (Nordmann et al., 2021), *E. coli* JJ1887 plasmid pJJ1887-5 (Johnson et al., 2016) and *E. coli* strain ARL09/232 plasmid pCO_Eco4457-3 (Olivera and Rakonjac, 2020). The AMR genes for both modules are shown in red. Metadata of the isolates and their IncFIB-IncFII plasmids are given in Supplementary Table S4. **(B)** Linear organization of the 24 kb bla_NDM-5_ accessory module in p10044824_1. A 288 bp remnant of IS*Aba125* located upstream of the *bla*_NDM-5_ gene carry an additional promoter for *bla*_NDM-5_. **(C)** Linear organization of the 14 kb accessory module in p10044824_1. IS*Ecp1* carries an additional promoter for *bla*_CTX-M-15_ gene. The IncFIB-IncFII plasmids in the three Hungarian *E. coli* isolates were identical, therefore, only plasmid p10044824_1 of *E. coli* strain M2021_10044824_1_E is shown in **(A)**. Similarly, the two MDR regions presented in **(B)** and **(C)** are identical among the three isolates, thus the regions in strain M2021_10044824_1_E only are presented.

Hybrid assembly of the isolates yielded complete closed genomes comprising a single circular chromosome (∼5.23 Mb, 50.5% GC) for strains M2021_10044802_2_E and M2021_10044824_1_E, and three plasmids for strains M2021_10044802_2_E and M2021_10043982_E, and two plasmids for strain M2021_10044824_1_E (lacking IncX4 replicon gene) (Table 2). The *bla*_NDM-5_-harboring plasmids (p10044824_1, p10044802_1, and p10043982_1; average size 132,009 bp, 50.74% GC) were identified as highly similar hybrid IncFII-IncFIB conjugative plasmids (FIB-32:FII-36 by pMLST) carrying an identical multidrug resistance (MDR) backbone, which included *bla*_NDM-5_, *bla*_CTX-M-15_, *bla*_TEM-1B_, *dfrA12*, complete *mph*(A) operon, *qepA4*, *sul1*, *tet*(B), and *aadA2*, along with complete *tra* and *trb* conjugal transfer gene clusters (Table 2; Figure 2A). The remaining plasmids consisted of a smaller plasmid of incompatibility group IncX4 (∼33 kb) and a phage-like p0111 plasmid (∼96 kb), both devoid of AMR determinants (Table 2).

**TABLE 2:** Plasmid content and AMR gene/mutations distribution in the hybrid assemblies of the three *bla*_NDM-5_-producing *E. coli* ST405 isolates from retail meat.

Isolate ID	M2021_10044802_2_E (beef)	M2021_10044824_1_E (pork)^a^	M2021_10043982_E (beef)
Plasmid replicon genes (% identity to the plasmidfinder db)	IncFIB (99%) IncFII (96%) IncX4 (97%) p0111 (99%)	IncFIB (99.22%) IncFII (96.18%) p0111 (98.64%)	IncFIB (99.22%) IncFII (96.18%) IncX4 (98.88%) p0111 (98.64%)
Plasmids (replicon genes, size, GC content)	p10044802_1 (IncFIB, IncFII, 131,952 bp, GC% 50.74) p10044802_2 (IncX4, 32,904 bp) Phage-like p10044802_3 (p0111, 96,750 bp)	p10044824_1 (IncFIB, IncFII, 132,041 bp, GC% 50.74) Phage-like p10044824_2 (p0111, 96,673 bp)	p10043982_1 (IncFIB, IncFII, 132,035 bp, GC% 50.74) p10043982_2 (IncX4, 32,930 bp) Phage-like p10043982_3 (p0111, 96,129 bp)
AMR genes	p10044802_1 (*bla*_CTX-M-15_, *bla*_NDM-5_, *bla*_TEM-1B_, *dfrA12, mph*(A), *qepA4*, *sul1*, *tet*(B), *aadA2*) Chromosome (*bla*_CTX-M-15_, *tet*(B))	p10044824_1 (*bla*_CTX-M-15_, *bla*_NDM-5_, *bla*_TEM-1B_, *dfrA12, mph*(A), *qepA4*, *sul1*, *tet*(B), *aadA2*) Chromosome (*bla*_CTX-M-15_, *tet*(B))	p10043982_1 (*bla*_CTX-M-15_, *bla*_NDM-5_, *bla*_TEM-1B_, *dfrA12, mph*(A), *qepA4*, *sul1*, *tet*(B*), aadA2*) Chromosome (−)

^a^ In this isolate, IncX4 replicon gene was only detected in the Illumina trimmed raw reads, but not in the Nanopore trimmed raw reads or the final assemblies.

Comparative genomic analysis of p10044824_1 against six representative IncFIB-IncFII plasmids from the United States (unnamed1, pJJ1887-5), Japan (pKY1497_1, pFUJ80154-1), Germany (pS253-NDM5) and New Zealand (pCO_Eco4457-3) revealed a mosaic structure with conserved backbone regions and variable accessory modules, illustrating the diversity of resistance gene cargo on globally disseminated IncF plasmids (Figure 2A; Supplementary Table S4). Detailed structural analysis of p10044824_1 identified two discrete accessory resistance modules: a 24 kbp multidrug resistance (MDR) module and a 14 kbp resistance island (Figures 2B,C). The 24 kbp MDR module comprised four functional units: (i) a class 1 integron (*intI1*) carrying *qepA4*, *dfrA12*, *aadA2*, and *sul1* gene cassettes; (ii) a *bla*_NDM-5_ region associated with a truncated IS*Aba125* element harboring an additional promoter sequence; (iii) a complete *mph*(A) module flanked by IS*26* and IS*6100* and containing *mph*(A), *mrx*, and *mph*(A)R; and (iv) a truncated Tn*3* transposon (Tn*2*) carrying *bla*_TEM-1B_ (Figure 2B). The 14 kbp accessory module contained *tet*(B) and *bla*_CTX-M-15_ genes, the latter associated with an IS*Ecp1* insertion sequence providing an additional promoter for *bla*_CTX-M-15_ expression (Figure 2C). Chromosomal copies of *bla*_CTX-M-15_ and *tet*(B) were additionally detected on the chromosome in M2021_10044802_2_E and M2021_10044824_1_E, indicating gene duplication events (Table 2).

Conjugation experiments using M2021_10044802_2_E as donor successfully transferred both IncFII-IncFIB (p10044802_1) and IncX4 (p10044802_2) plasmids to the rifampicin-resistant recipient *E. coli* K12 20R764. TCs exhibited antimicrobial resistance profiles concordant with the genetic content of p10044802_1, including resistance to azithromycin, β-lactams (carbapenems, extended-spectrum cephalosporins, ampicillin, temocillin), ciprofloxacin (*qepA*-mediated), trimethoprim, and tetracycline (Table 1). Notably, despite carrying *sul1*, TCs remained phenotypically susceptible to sulfamethoxazole (MIC ≤8 mg/L), and imipenem and meropenem MIC values were reduced in TCs compared to the donor strain (2 vs. 8–16 mg/L), suggesting host-dependent modulation of carbapenem resistance expression or additional chromosomal factors contributing to the donor’s higher-level resistance phenotype (Table 1).

## 4 Discussion

The detection of clonally related *bla*_NDM-5_-producing *E. coli* ST405 isolates in retail beef and pork in Hungary represents a significant One Health concern, particularly given the close genetic relationship (HC2-172694) observed with ST405 isolates from human clinical specimens in Australia and the United Kingdom sharing identical AMR profiles. This finding demonstrates international dissemination of this carbapenem-resistant clone across multiple continents and strongly suggests human contamination of the food chain (European Centre for Disease Prevention and Control, 2023; Linkevicius et al., 2023). This is consistent with findings from Italy where *bla*_OXA-48_-harboring *E. coli* in meat were traced back to human sources (Carfora et al., 2022), and similar to recent reports from Czechia, Spain, and other European countries (Hans et al., 2023) where carbapenemase-producing Enterobacterales have been detected in food-producing animals and their derived meat. ST405 is recognized as a globally distributed high-risk clone and a well-established human uropathogenic sequence type increasingly associated with multidrug resistance, *bla*_NDM-5_ carbapenemase production, and enhanced virulence (Chowdhury et al., 2019; Xia et al., 2024; Wang et al., 2025). A comprehensive global genomic analysis of 1,774 *bla*_NDM_-producing *E. coli* from 43 countries confirmed ST405 as one of the major epidemic clones, with 74.1% carrying *bla*_NDM-5_, predominantly on IncFII and IncFIB plasmids (Xia et al., 2024).

The European Centre for Disease Prevention and Control (ECDC) has documented a deteriorating epidemiological situation regarding carbapenemase-producing Enterobacterales in the EU/EEA, with increasing detection of high-risk lineages of *E. coli* carrying carbapenemase genes, including ST167, ST361, ST405, ST410, and ST648 carrying *bla*_NDM-5_, posing risks for community transmission (European Centre for Disease Prevention and Control, 2025). Hungary reported two *bla*_NDM-5_-producing *E. coli* cases in 2020 (European Centre for Disease Prevention and Control, 2023), and the current detection in retail meat from 2021 underscores the continued circulation of these strains. The three Hungarian ST405 O102:H6 isolates also harbored an extensive repertoire of 72-76 virulence-associated genes from the VFDB database, including complete operons for multiple iron acquisition systems (the yersiniabactin (*ybt*), enterobactin (*ent*/*fep*) and heme uptake *chuA*–*chuS* loci), diverse fimbrial adhesins (type 1 fimbriae *fim* genes, P fimbriae *pap* genes, and the *ecp* operon for *E. coli* common pilus), and Type III secretion system T3SS) effectors, among others. This enhanced virulence potential is consistent with recent comparative genomic studies demonstrating that ST405 clones harbor significantly more virulence genes than other carbapenem-resistant *E. coli* clones (Wang et al., 2025) and exhibit enhanced virulence in animal models (Peri et al., 2020; Wang et al., 2025). Additionally, all three isolates carried *eaeX*, an invasiveness-associated gene reported in 79.2% of ST405 isolates but absent from other carbapenem-resistant *E. coli* clones (Chowdhury et al., 2019), confirming the genetic signature typical of this high-risk sequence type.

While ST405 is primarily a human-adapted uropathogenic clone (Chowdhury et al., 2019), the *bla*_NDM-5_-harbouring IncFII-IncFIB conjugative plasmids (FIB-32:FII-36) identified in this study demonstrate the capacity for horizontal gene transfer driving antimicrobial resistance dissemination across host species and geographical boundaries, with potential spread through trade and food chain pathways (Fu et al., 2024). Our conjugation experiments confirmed successful transfer of these plasmids to recipient *E. coli* K12, with TCs exhibiting resistance phenotypes concordant with the plasmid-encoded multidrug resistance backbone comprising *bla*_NDM-5_, *bla*_CTX-M-15_, *bla*_TEM-1B_, *dfrA12*, *mph*(A), *qepA4*, *sul1*, *tet(B)*, and *aadA2*. This co-occurrence of carbapenemase genes with determinants conferring resistance to other critically important antibiotics, including extended-spectrum β-lactamases, plasmid-mediated quinolone resistance, and macrolide resistance, substantially increases the risk for co-selection, maintenance, and dissemination in both human and animal reservoirs (Sun et al., 2016; Bortolaia et al., 2021; Xia et al., 2024), drastically limiting therapeutic options for life-threatening infections.

The genetic context of *bla*_NDM-5_ [ΔIS*Aba125*-*bla*_NDM-5_-*ble*_MBL_-*trpT*-*dsb*-IS*26*] is highly conserved and has been previously reported in both IncF-type and IncX3 plasmids globally (Chakraborty et al., 2021; Turton et al., 2022), with IncFII (65.6%) and IncFIB (65.2%) representing the most prevalent plasmid replicons in *bla*_NDM_-carrying *E. coli* worldwide (Xia et al., 2024). Comparative analysis with representative IncFIB-IncFII plasmids from the United States, Japan, Germany, and New Zealand revealed a mosaic structure with conserved backbone regions and variable accessory modules carrying diverse resistance gene cargo. The modular organization identified in our structural analysis, comprising discrete resistance islands with class 1 integrons, IS-mediated insertions (particularly through IS*26*), and recombinant regions, demonstrates the capacity of IncF plasmids to accumulate and recombine resistance determinants through mobile genetic element-mediated events, underscoring their plasticity and evolutionary potential (Xia et al., 2024). These findings indicate the capacity of such plasmids to disseminate across the livestock production chain from breeding facilities to slaughterhouses and retail markets, posing ongoing risks for carbapenemase spread in food systems (Fu et al., 2024).

Ideally, findings of CPEs in food-producing animals should be followed up and investigated thoroughly with high sensitivity and specificity isolation protocols, including carbapenem-selective enrichment methods currently being developed in the EFSA project: Data Generation on Carbapenemase-producing Enterobacterales (CPEs) in the food chain in the EU/EFTA “Carba-R-ales” (Espinosa Gongora et al., 2024). Investigations of the source of introduction, persistence and transmission in farm and food-processing environments are critical for developing strategies to prevent CPEs spread to humans through the food chain and improve food safety (Irrgang et al., 2020; Carfora et al., 2022). While epidemiological investigations were not undertaken in Hungary and no source-tracing information was available, the most plausible origin of the *bla*_NDM-5_-carrying *E. coli* ST405 clone in retail meat is human contamination, as ST405 is a recognised human uropathogenic ST with documented fecal carriage and environmental contamination potential (Chowdhury et al., 2019; Wang et al., 2025).

## 5 Conclusion

The emerging resistance to carbapenems in the primary production of animals in several European countries, including recent findings of the *bla*_NDM-5_-carrying *E. coli* in Hungary, Italy, Czechia, Spain and other European countries, demands immediate attention from risk managers, politicians and policymakers. The detection of three clonally related *bla*_NDM-5_-producing *E. coli* ST405 isolates (serotype O102:H6) in retail beef and pork in Hungary, combined with their close genetic relationship to human clinical isolates from Australia and the United Kingdom sharing the same AMR profiles, underscores the international dissemination of this high-risk clone and demonstrates the potential for bidirectional transmission between human and food chain reservoirs, with the human-adapted nature of ST405 suggesting contamination from human sources into the food chain. The demonstrated horizontal transferability of IncFII-IncFIB hybrid plasmids, coupled with the co-carriage of resistance determinants against critically important antibiotics (including *qepA4*, *bla*_CTX-M-15_), further amplifies the public health risk by enabling resistance dissemination across bacterial populations in both human and animal reservoirs. The extensive repertoire of virulence factors detected in these isolates, including multiple iron acquisition systems and Type III secretion system effectors characteristic of extraintestinal pathogenic *E. coli*, compounds the clinical risk beyond antimicrobial resistance alone. Collectively, these findings pose a significant threat comparable to the widespread resistance to fluoroquinolones and cephalosporins, potentially rendering carbapenems, antibiotics of last resort, ineffective for treating severe infections caused by MDR bacteria.

Moreover, continuous CPE monitoring, comprehensive strain characterization through whole-genome sequencing, and rigorous follow-up epidemiological investigations are critical to elucidate their origin, transmission routes, and persistence factors along the food chain. Specifically, source-tracing studies linking retail meat contamination to farm environments and food-processing facilities are essential. These efforts must be complemented by enhanced carbapenem-selective enrichment protocols currently under development in EFSA’s “Carba-R-ales” project to improve detection sensitivity.

The convergence of genomic, epidemiological, and phenotypic evidence presented in this study underscores the urgent need for integrated One Health surveillance frameworks that span human clinical settings, livestock production systems, and retail food chains. Only through sustained global collaboration, harmonized surveillance strategies, stringent antimicrobial stewardship, and evidence-based interventions can we effectively mitigate the impact of carbapenemase-producing Enterobacterales and preserve the efficacy of last-resort antibiotics for future generations.

## Data Availability

The datasets presented in this study can be found in online repositories. The names of the repository/repositories and accession number(s) can be found in the article/Supplementary Material.

## References

[B1] Alikhan N.-F. Petty N. K. Ben Zakour N. L. Beatson S. A. (2011). BLAST ring image generator (BRIG): simple prokaryote genome comparisons. BMC Genomics 12, 402. 10.1186/1471-2164-12-402 21824423 PMC3163573

[B2] Andrews S. (2010). FastQC. A quality control tool for high throughput sequence data. Available online at: http://www.bioinformatics.babraham.ac.uk/projects/fastqc/ (Accessed November 19, 2023).

[B3] Bonomo R. A. Burd E. M. Conly J. Limbago B. M. Poirel L. Segre J. A. (2018). Carbapenemase-producing organisms: a global scourge. Clin. Infect. Dis. 66, 1290–1297. 10.1093/cid/cix893 29165604 PMC5884739

[B4] Bortolaia V. Kaas R. S. Ruppe E. Roberts M. C. Schwarz S. Cattoir V. (2020). ResFinder 4.0 for predictions of phenotypes from genotypes. J. Antimicrob. Chemother. 75, 3491–3500. 10.1093/jac/dkaa345 32780112 PMC7662176

[B5] Bortolaia V. Ronco T. Romascu L. Nicorescu I. Milita N. M. Vaduva A. M. (2021). Co-localization of carbapenem (*bla* OXA-162) and colistin (*mcr-1*) resistance genes on a transferable IncHI2 plasmid in *Escherichia coli* of chicken origin. J. Antimicrob. Chemother. 76, 3063–3065. 10.1093/jac/dkab285 34392339 PMC8521400

[B6] Bushnell B. Rood J. Singer E. (2017). BBMerge – accurate paired shotgun read merging via overlap. PLoS One 12, e0185056. 10.1371/journal.pone.0185056 29073143 PMC5657622

[B7] Carattoli A. Zankari E. García-Fernández A. Voldby Larsen M. Lund O. Villa L. (2014). *In silico* detection and typing of plasmids using PlasmidFinder and plasmid Multilocus sequence typing. Antimicrob. Agents Chemother. 58, 3895–3903. 10.1128/AAC.02412-14 24777092 PMC4068535

[B8] Carfora V. Diaconu E. L. Ianzano A. Di Matteo P. Amoruso R. Dell’Aira E. (2022). The hazard of carbapenemase (OXA-181)-producing *Escherichia coli* spreading in pig and veal calf holdings in Italy in the genomics era: risk of spill over and spill back between humans and animals. Front. Microbiol. 13, 1016895. 10.3389/fmicb.2022.1016895 36466661 PMC9712188

[B9] Chakraborty T. Sadek M. Yao Y. Imirzalioglu C. Stephan R. Poirel L. (2021). Cross-border emergence of *Escherichia coli* producing the carbapenemase NDM-5 in Switzerland and Germany. J. Clin. Microbiol. 59, e02238-20. 10.1128/JCM.02238-20 33361340 PMC8106709

[B10] Chowdhury P. McKinnon J. Liu M. Djordjevic S. P. (2019). Multidrug resistant uropathogenic *Escherichia coli* ST405 with a novel, composite IS26 transposon in a unique chromosomal location. Front. Microbiol. 9, 3212. 10.3389/fmicb.2018.03212 30671039 PMC6331395

[B11] De Coster W. Rademakers R. (2023). NanoPack2: population-scale evaluation of long-read sequencing data. Bioinformatics 39, btad311. 10.1093/bioinformatics/btad311 37171891 PMC10196664

[B12] ECDC (2023). The european union summary report on antimicrobial resistance in zoonotic and indicator bacteria from humans, animals and food in 2020/2021. EFSA J. 21 (3), e07867. 10.2903/j.efsa.2023.7867 36891283 PMC9987209

[B13] Element S. J. Moran R. A. Beattie E. Hall R. J. Van Schaik W. Buckner M. M. C. (2023). Growth in a biofilm promotes conjugation of a *bla* _NDM-1_-bearing plasmid between *Klebsiella pneumoniae* strains. mSphere 8. 10.1128/msphere.00170-23 37417759 PMC10449501

[B14] Espinosa Gongora C. Hendriksen R. S. Kjeldgaard J. S. (2024). Carba-R-ales: data generation on carbapenemase-producing Enterobacterales (CPEs) in the food chain in the EU/EFTA. Available online at: https://orbit.dtu.dk/en/projects/carba-r-ales-data-generation-on-carbapenemase-producing-enterobac/ (Accessed November 19, 2023).

[B15] European Centre for Disease Prevention and Control. (2023). Increase in *Escherichia coli* isolates carrying blaNDM-5 in the european union/european economic area, 2012–2022. Stockholm: European Centre for Disease Prevention and Control. Available online at: https://data.europa.eu/doi/10.2900/72700 (Accessed October 26, 2025).

[B16] European Centre for Disease Prevention and Control. (2025). Carbapenem-resistant Enterobacterales: third update. Publications Office. Available online at: https://data.europa.eu/doi/10.2900/8752612 (Accessed December 22, 2025).

[B17] European Food Safety Authority and European Centre for Disease Prevention and ControlEuropean Centre for Disease Prevention and Control (2025). The european union summary report on antimicrobial resistance in zoonotic and indicator bacteria from humans, animals and food in 2022–2023. EFSA J. 23 (3), e9237. 10.2903/j.efsa.2025.9237 PMC11880826 40046901

[B18] European Food Safety Authority, (EFSA) Amore G. Beloeil P. Fierro R. G. Guerra B. Papanikolaou A. (2021). Manual for reporting 2021 antimicrobial resistance data within the framework of directive 2003/99/EC and decision 2020/1729/EU. EFSA supporting publication 2021:EN-6652. 10.2903/sp.efsa.2021.EN-6652

[B19] Fu B. Xu J. Yin D. Sun C. Liu D. Zhai W. (2024). Transmission of *bla* _NDM_ in enterobacteriaceae among animals, food and human. Emerg. Microbes & Infect. 13, 2337678. 10.1080/22221751.2024.2337678 38629492 PMC11034458

[B20] Gurevich A. Saveliev V. Vyahhi N. Tesler G. (2013). QUAST: quality assessment tool for genome assemblies. Bioinformatics 29, 1072–1075. 10.1093/bioinformatics/btt086 23422339 PMC3624806

[B21] Hans J. B. Pfennigwerth N. Neumann B. Pfeifer Y. Fischer M. A. Eisfeld J. (2023). Molecular surveillance reveals the emergence and dissemination of NDM-5-producing *Escherichia coli* high-risk clones in Germany, 2013 to 2019. Eurosurveillance 28, 2200509. 10.2807/1560-7917.ES.2023.28.10.2200509 36892470 PMC9999457

[B22] Hendriksen R. S. Cavaco L. M. Guerra B. Bortolaia V. Agersø Y. Svendsen C. A. (2023). Evaluation and validation of laboratory procedures for the surveillance of ESBL-AmpC-and carbapenemase-producing *Escherichia coli* from fresh meat and caecal samples. Front. Microbiol. 14, 1229542. 10.3389/fmicb.2023.1229542 37621395 PMC10445139

[B23] Irrgang A. Tausch S. H. Pauly N. Grobbel M. Kaesbohrer A. Hammerl J. A. (2020). First detection of GES-5-producing *Escherichia coli* from livestock-an increasing diversity of carbapenemases recognized from German pig production. Microorganisms 8, 1593. 10.3390/microorganisms8101593 33081194 PMC7602714

[B24] Ivanova M. Ovsepian A. Leekitcharoenphon P. Seyfarth A. M. Mordhorst H. Otani S. (2024). Azithromycin resistance in *Escherichia coli* and *Salmonella* from food-producing animals and meat in Europe. J. Antimicrob. Chemother. 79, 1657–1667. 10.1093/jac/dkae161 38775752 PMC11215539

[B25] Joensen K. G. Tetzschner A. M. M. Iguchi A. Aarestrup F. M. Scheutz F. (2015). Rapid and easy *in silico* serotyping of *Escherichia coli* isolates by use of whole-genome sequencing data. J. Clin. Microbiol. 53, 2410–2426. 10.1128/JCM.00008-15 25972421 PMC4508402

[B26] Johnson T. J. Aziz M. Liu C. M. Sokurenko E. Kisiela D. I. Paul S. (2016). Complete genome sequence of a CTX-M-15-producing *Escherichia coli* strain from the H30Rx subclone of sequence type 131 from a patient with recurrent urinary tract infections, closely related to a lethal urosepsis isolate from the patient’s sister. Genome announc. 4, e00334-16. 10.1128/genomeA.00334-16 27174264 PMC4866839

[B27] Kaas R. S. Leekitcharoenphon P. Aarestrup F. M. Lund O. (2014). Solving the problem of comparing whole bacterial genomes across different sequencing platforms. PLoS ONE 9, e104984. 10.1371/journal.pone.0104984 25110940 PMC4128722

[B28] Kolmogorov M. Yuan J. Lin Y. Pevzner P. A. (2019). Assembly of long, error-prone reads using repeat graphs. Nat. Biotechnol. 37, 540–546. 10.1038/s41587-019-0072-8 30936562

[B29] Letunic I. Bork P. (2019). Interactive tree of life (iTOL) v4: recent updates and new developments. Nucleic Acids Res. 47, 256–259. 10.1093/nar/gkz239 30931475 PMC6602468

[B30] Linkevicius M. Bonnin R. A. Alm E. Svartström O. Apfalter P. Hartl R. (2023). Rapid cross-border emergence of NDM-5-producing *Escherichia coli* in the european union/european economic area. Eurosurveillance 28, 2300209. 10.2807/1560-7917.ES.2023.28.19.2300209 37166762 PMC10176832

[B31] Liu B. Zheng D. Zhou S. Chen L. Yang J. (2022). VFDB 2022: a general classification scheme for bacterial virulence factors. Nucleic Acids Res. 50, D912–D917. 10.1093/nar/gkab1107 34850947 PMC8728188

[B32] Minh B. Q. Schmidt H. A. Chernomor O. Schrempf D. Woodhams M. D. Von Haeseler A. (2020). IQ-TREE 2: new models and efficient methods for phylogenetic inference in the genomic era. Mol. Biol. Evol. 37, 1530–1534. 10.1093/molbev/msaa015 32011700 PMC7182206

[B33] Mujahid F. Rasool M. H. Shafiq M. Aslam B. Khurshid M. (2024). Emergence of carbapenem-resistant uropathogenic *Escherichia coli* (ST405 and ST167) strains carrying *bla* _CTX-M-15_, *bla* _NDM-5_ and diverse virulence factors in hospitalized patients. Pathogens 13, 964. 10.3390/pathogens13110964 39599517 PMC11597634

[B34] Nordmann P. Yao Y. Falgenhauer L. Sadek M. Imirzalioglu C. Chakraborty T. (2021). Recent emergence of aztreonam-avibactam resistance in NDM and OXA-48 carbapenemase-producing *Escherichia coli* in Germany. Antimicrob. Agents Chemother. 65, e01090-21. 10.1128/AAC.01090-21 34424048 PMC8522741

[B35] Olivera C. Rakonjac J. (2020). Complete genome assembly of a multidrug-resistant New Delhi metallo-β-lactamase 1 (NDM-1)-producing *Escherichia coli* human isolate from a New Zealand hospital. Microbiol. Resour. Announc 9. 10.1128/MRA.00780-20 32816984 PMC7441242

[B36] Peri A. M. Piazza A. De Zan V. Carugati M. Muscatello A. Comandatore F. (2020). Autochthonous ST405 NDM-5 producing *Escherichia coli* causing fatal sepsis in Northern Italy. Int. J. Antimicrob. Agents 55, 105953. 10.1016/j.ijantimicag.2020.105953 32289435

[B37] Schwengers O. Jelonek L. Dieckmann M. A. Beyvers S. Blom J. Goesmann A. (2021). Bakta: rapid and standardized annotation of bacterial genomes *via* alignment-free sequence identification. Microb. Genomics 7, 000685. 10.1099/mgen.0.000685 34739369 PMC8743544

[B38] Sherry N. L. Lane C. R. Kwong J. C. Schultz M. Sait M. Stevens K. (2019). Genomics for molecular epidemiology and detecting transmission of carbapenemase-producing *Enterobacterales* in Victoria, Australia, 2012 to 2016. J. Clin. Microbiol. 57, e00573-19. 10.1128/JCM.00573-19 31315956 PMC6711911

[B39] Siguier P. Perochon J. Lestrade L. Mahillon J. Chandler M. (2006). ISfinder: the reference centre for bacterial insertion sequences. Nucleic Acids Res. 34, D32–D36. 10.1093/nar/gkj014 16381877 PMC1347377

[B40] Sivarajan V. Ganesh A. V. Subramani P. Ganesapandi P. Sivanandan R. N. Prakash S. (2025). Prevalence and genomic insights of carbapenem resistant and ESBL producing multidrug resistant *Escherichia coli* in urinary tract infections. Sci. Rep. 15, 2541. 10.1038/s41598-024-84754-w 39833199 PMC11747333

[B41] Sun J. Yang R.-S. Zhang Q. Feng Y. Fang L.-X. Xia J. (2016). Co-transfer of *bla* _NDM-5_ and *mcr-1* by an IncX3–X4 hybrid plasmid in *Escherichia coli* . Nat. Microbiol. 1, 16176. 10.1038/nmicrobiol.2016.176 27668643

[B42] Takayama Y. Sekizuka T. Matsui H. Adachi Y. Eda R. Nihonyanagi S. (2020). Characterization of the IncFII-IncFIB(pB171) plasmid carrying bla_NDM-5_ in *Escherichia coli* ST405 clinical isolate in Japan. IDR 13, 561–566. 10.2147/IDR.S232943 32110066 PMC7035895

[B43] Turton J. F. Pike R. Perry C. Jenkins C. Turton J. A. Meunier D. (2022). Wide distribution of *Escherichia coli* carrying IncF plasmids containing *bla* _NDM-5_ and *rmtB* resistance genes from hospitalized patients in England. J. Med. Microbiol. 71, 1–9. 10.1099/jmm.0.001569 35925786

[B44] Wang M. Zhang Z. Sun Z. Wang X. Zhu J. Jiang M. (2025). The emergence of highly resistant and hypervirulent *Escherichia coli* ST405 clone in a tertiary hospital over 8 years. Emerg. Microbes & Infect. 14, 2479048. 10.1080/22221751.2025.2479048 40071947 PMC11934165

[B45] Wick R. R. Holt K. E. (2022). Polypolish: short-read polishing of long-read bacterial genome assemblies. PLoS Comput. Biol. 18, e1009802. 10.1371/journal.pcbi.1009802 35073327 PMC8812927

[B46] Xia C. Yan R. Liu C. Zhai J. Zheng J. Chen W. (2024). Epidemiological and genomic characteristics of global *bla* _NDM_-carrying *Escherichia coli* . Ann. Clin. Microbiol. Antimicrob. 23, 58. 10.1186/s12941-024-00719-x 38907245 PMC11193274

[B47] Zankari E. Allesøe R. Joensen K. G. Cavaco L. M. Lund O. Aarestrup F. M. (2017). PointFinder: a novel web tool for WGS-based detection of antimicrobial resistance associated with chromosomal point mutations in bacterial pathogens. J. Antimicrob. Chemother. 72, 2764–2768. 10.1093/jac/dkx217 29091202 PMC5890747

[B48] Zhang Z. Guo H. Li X. Li W. Yang G. Ni W. (2021). Genetic diversity and characteristics of *bla* _NDM_-positive plasmids in *Escherichia coli* . Front. Microbiol. 12, 729952. 10.3389/fmicb.2021.729952 34867846 PMC8636099

