# Correction: Human-associated NDM-5-producing multidrug-resistant *Escherichia coli* detected in retail beef and pork in Hungary, 2021

**DOI:** 10.3389/fbinf.2026.1842628

**Published:** 2026-04-13

**Authors:** Frontiers Production Office

**Affiliations:** Frontiers Media SA, Lausanne, Switzerland

**Keywords:** blaNDM-5, carbapenemase-producing Enterobacterales, *Escherichia coli* ST405, horizontal gene transfer, IncFII-IncFIB plasmid, multidrug resistance, one health surveillance

Editor Manoj K. Sekhwal was erroneously spelled as Manoj Kumar. The correct spelling is **Manoj K. Sekhwal**.

The original article has been updated.

