# Supplementary material for: Human-associated NDM-5-producing multidrug-resistant Escherichia coli detected in retail beef and pork in Hungary, 2021
Source: Front Bioinform. 2026 Mar 24;6:1793862. doi: 10.3389/fbinf.2026.1793862 (PMC13053247; doi:10.3389/fbinf.2026.1793862)
Supplement: Supplementary file 1 [file Supplementaryfile1.docx]

Supplementary Material

# Supplementary Data

# Supplementary Figures

**Figure S1.** Distributions of read length versus read quality for the three *E. coli* strains (M2021_10043982_E, M2021_10044802_2_E, and M2021_10044824_1_E) before and after quality trimming with Filtlong.

# Supplementary Tables

**Table S1.** Summary statistics of the polished Nanopore assemblies generated using QUAST.

| **Statistic** | **10043982_polished_assembly*** | **10044802_polished_assembly** | **10044824_polished_assembly** |
| --- | --- | --- | --- |
| # contigs (>= 25000 bp) | 23 | 4 | 3 |
| # contigs (>= 50000 bp) | 19 | 3 | 3 |
| Total length (>= 25000 bp) | 4342519 | 5490960 | 5458131 |
| Total length (>= 50000 bp) | 4198420 | 5458035 | 5458131 |
| # contigs | 23 | 4 | 3 |
| Largest contig | 786189 | 5229313 | 5229423 |
| Total length | 4342519 | 5490960 | 5458131 |
| GC (%) | 50.53 | 50.46 | 50.52 |
| N50 | 359570 | 5229313 | 5229423 |
| # N's per 100 kbp | 0 | 0 | 0 |
|  |  |  |  |
| *For isolate 10043982 plasmid DNA only (usually plasmid DNA is contaminated with chromosomal, thus the larger DNA size) | | | |

**Table S2.** Complete antimicrobial resistance profiles and associated genetic determinants of the three *bla*_NDM-5_-producing *E. coli* ST405 isolates and transconjugant TC_M2021-10044802/2-E.

|  | **D_M2021-10044802/2-E** | | | **TC_M2021-10044802/2-E^2^** | | | **M2021-10043982-E** | | | **M2021-10044824/1-E** | | |
| --- | --- | --- | --- | --- | --- | --- | --- | --- | --- | --- | --- | --- |
| **Antibiotic** | **AMR phenotype^1^** | **MIC (mg/L)** | **Genotype** | **AMR phenotype^1^** | **MIC (mg/L)** | **Genotype** | **AMR phenotype^1^** | **MIC (mg/L)** | **Genotype** | **AMR phenotype^1^** | **MIC (mg/L)** | **Genotype** |
| Amikacin | S | ≤4 | - | S | ≤4 | - | S | ≤4 | - | S | ≤4 | - |
| Ampicillin | R | >32 | *bla*_TEM-1B,_ *bla*_CTX-M-15,_ *bla*_NDM-5_ | **R** | **>32** | *bla*_TEM-1B,_ *bla*_CTX-M-15,_ *bla*_NDM-5_ | R | >32 | *bla*_TEM-1B,_ *bla*_CTX-M-15,_ *bla*_NDM-5_ | R | >32 | *bla*_TEM-1B,_ *bla*_CTX-M-15,_ *bla*_NDM-5_ |
| Azithromycin | R | 64 | *mph*(A) | **R** | **32** | *mph*(A) | R | 64 | *mph*(A) | R | 64 | *mph*(A) |
| Cefepime | R | >32 | *bla*_CTX-M-15,_ *bla*_NDM-5_ | **R** | **16** | *bla*_CTX-M-15,_ *bla*_NDM-5_ | R | >32 | *bla*_CTX-M-15,_ *bla*_NDM-5_ | R | >32 | *bla*_CTX-M-15,_ *bla*_NDM-5_ |
| Cefotaxime | R | >4 | *bla*_CTX-M-15,_ *bla*_NDM-5_ | **R** | **>4** | *bla*_CTX-M-15,_ *bla*_NDM-5_ | R | >4 | *bla*_CTX-M-15,_ *bla*_NDM-5_ | R | >4 | *bla*_CTX-M-15,_ *bla*_NDM-5_ |
| Cefotaxime/Clavulanic acid | R | >64/4 | *bla*_CTX-M-15,_ *bla*_NDM-5_ | **R** | **>64/4** | *bla*_CTX-M-15,_ *bla*_NDM-5_ | R | >64 | *bla*_NDM-5_ | R | >64 | *bla*_CTX-M-15,_ *bla*_NDM-5_ |
| Ceftazidime | R | >8 | *bla*_CTX-M-15,_ *bla*_NDM-5_ | **R** | **>8** | *bla*_CTX-M-15,_ *bla*_NDM-5_ | R | >8 | *bla*_CTX-M-15,_ *bla*_NDM-5_ | R | >8 | *bla*_CTX-M-15,_ *bla*_NDM-5_ |
| Ceftazidime/Clavulanic acid | R | >128/4 | *bla*_CTX-M-15,_ *bla*_NDM-5_ | **R** | **>128/4** | *bla*_CTX-M-15,_ *bla*_NDM-5_ |  | >128 | *bla*_NDM-5_ | R | >128 | *bla*_CTX-M-15,_ *bla*_NDM-5_ |
| Chloramphenicol | S | ≤8 | - | S | ≤8 | - | S | ≤8 | - | S | ≤8 | - |
| Ciprofloxacin | R | >8 | *qepA, gyrA* (p.S83L), *gyrA* (p.D87N), *parE* (p.S458A), *parC* (p.S80I) | **R** | **0.12** | ***qepA*** | R | >8 | *qepA, gyrA* (p.S83L), *gyrA* (p.D87N), *parE* (p.S458A), *parC* (p.S80I) | R | >8 | *qepA, gyrA* (p.S83L), *gyrA* (p.D87N), *parE* (p.S458A), *parC* (p.S80I) |
| Cefoxitin | R | >64 | *bla*_NDM-5_ | **R** | **>64** | ***bla*_NDM-5_** | R | >64 | *bla*_NDM-5_ | R | >64 | *bla*_NDM-5_ |
| Colistin | S | ≤1 | - | S | ≤1 | - | S | ≤1 | - | S | ≤1 | - |
| Ertapenem | R | >2 | *bla*_NDM-5_ | **R** | **>2** | *bla*_NDM-5_ | R | >2 | *bla*_NDM-5_ | R | >2 | *bla*_NDM-5_ |
| Gentamycin | S | ≤0.5 | - | S | ≤0.5 | - | S | ≤0.5 | - | S | ≤0.5 | - |
| Imipenem | R | 8 | *bla*_NDM-5_ | **R** | **2** | *bla*_NDM-5_ | R | 8 | *bla*_NDM-5_ | R | 16 | *bla*_NDM-5_ |
| Meropenem | R | 16 | *bla*_NDM-5_ | **R** | **2** | *bla*_NDM-5_ | R | 16 | *bl*a_NDM-5_ | R | 16 | *bla*_NDM-5_ |
| Nalidixic acid | R | >64 | *gyrA* (p.S83L), *gyrA* (p.D87N), *parE* (p.S458A), *parC* (p.S80I) | S | ≤4 | - | R | >64 | *gyrA* (p.S83L), *gyrA* (p.D87N), *parE* (p.S458A), *parC* (p.S80I) | R | >64 | *gyrA* (p.S83L), *gyrA* (p.D87N), *parE* (p.S458A), *parC* (p.S80I) |
| Sulfamethoxazole | R | 512 | *sul1* (99.76% coverage) | S^3^ | ≤8 | *sul1* | R | >512 | *sul1* | R | 512 | *sul1* |
| Tetracycline | R | >32 | *tet*(B) | **R** | **>32** | *tet*(B) | R | >32 | *tet*(B) | R | >32 | *tet*(B) |
| Tigecycline | S | ≤0.25 | - | S | ≤0.25 | - | S | ≤0.25 | - | S | ≤0.25 | - |
| Trimethoprim | R | 16 | *dfrA12* | **R** | **>16** | *dfrA12* | R | 16 | *dfrA12* | R | >16 | *dfrA12* |
| Temocillin | R | >128 | *bla*_NDM-5_ | **R** | **32** | *bla*_NDM-5_ | R | >128 | *bla*_NDM-5_ | R | 128 | *bla*_NDM-5_ |

-: No AMR genes or mutations detected.

^1^ The antimicrobial susceptibility profiles were tested by the broth microdilution assay using EUVSEC2 and EUVSEC3 Sensititre™ panels (Thermo Fisher Scientific, MA, USA) according to the manufacturer’s instructions. The obtained MIC values were interpreted according to EUCAST epidemiological cut-off values (http://eucast.org/) or EFSA-defined surveillance ECOFFs (19).

^2^ The recipient strain *E. coli* K12 20R764 (ST10, rifampicin-resistant) exhibited susceptibility to all antimicrobials tested in this study, with MIC values of ≤0.015 mg/L for ertapenem; ≤0.03 mg/L for ciprofloxacin and meropenem; ≤0.06 mg/L for cefepime; ≤0.12 mg/L for imipenem; ≤0.25 mg/L for cefotaxime, ceftazidime, trimethoprim, and tigecycline; ≤0.5 mg/L for gentamicin; ≤1 mg/L for colistin; ≤2 mg/L for tetracycline; ≤4 mg/L for amikacin, ampicillin, azithromycin, cefoxitin, and nalidixic acid; ≤8 mg/L for sulfamethoxazole and chloramphenicol; 8 mg/L for temocillin; ≤0.06/4 mg/L for cefotaxime/clavulanic acid; and ≤0.12/4 mg/L for ceftazidime/clavulanic acid. Bold typeface denotes antimicrobial resistance phenotypes in TCs that were successfully transferred from and correspond to those of the donor strain.

^3^ Despite carrying *sul1*, the TC repeatedly showed a MIC ≤ 8 mg/L.

**Table S3.** Virulence-associated genes identified in the three *E. coli* ST405 O102:H6 isolates. Virulence genes were detected in short-read (Illumina) assemblies using the Virulence Factor Database (VFDB) with minimum identity of 90% and minimum coverage of 90%. Genes are organized by their associated virulence factor systems and functional categories.

| **No.** | **Virulence Factor System** | **Gene** | **Product/Function** | **M2021_10044824_1_E** | **M2021_10043982_E** | **M2021_10044802_2_E** |
| --- | --- | --- | --- | --- | --- | --- |
| **Iron Acquisition Systems** | | | | | | |
| 1 | Yersiniabactin | *fyuA* | Yersiniabactin receptor | + | + | + |
| 2 | Yersiniabactin | *irp1* | Yersiniabactin biosynthetic protein | + | + | + |
| 3 | Yersiniabactin | *irp2* | Yersiniabactin biosynthetic protein | + | + | + |
| 4 | Yersiniabactin | *ybtA* | Transcriptional regulator | + | + | + |
| 5 | Yersiniabactin | *ybtE* | Siderophore biosynthetic protein | + | + | + |
| 6 | Yersiniabactin | *ybtP* | ABC transporter lipoprotein | + | + | + |
| 7 | Yersiniabactin | *ybtQ* | ABC transporter | + | + | + |
| 8 | Yersiniabactin | *ybtS* | Salicylate synthase | + | + | + |
| 9 | Yersiniabactin | *ybtT* | Biosynthetic protein | + | + | + |
| 10 | Yersiniabactin | *ybtU* | Biosynthetic protein | + | + | + |
| 11 | Yersiniabactin | *ybtX* | Signal transducer | + | + | + |
| 12 | Enterobactin | *entA* | Dihydroxybenzoate dehydrogenase | + | + | + |
| 13 | Enterobactin | *entB* | Isochorismatase | + | + | + |
| 14 | Enterobactin | *entC* | Isochorismate synthase 1 | + | + | + |
| 15 | Enterobactin | *entD* | Phosphopantetheinyl transferase | + | + | + |
| 16 | Enterobactin | *entE* | Dihydroxybenzoate-AMP ligase | + | + | + |
| 17 | Enterobactin | *entF* | Enterobactin synthase component | + | + | + |
| 18 | Enterobactin | *entS* | Enterobactin exporter | + | + | + |
| 19 | Enterobactin | *fepA* | Ferrienterobactin transporter | + | + | + |
| 20 | Enterobactin | *fepB* | ABC transporter periplasmic protein | + | + | + |
| 21 | Enterobactin | *fepC* | ABC transporter ATPase | + | + | + |
| 22 | Enterobactin | *fepD* | ABC transporter permease | + | + | + |
| 23 | Enterobactin | *fepG* | ABC transporter permease | + | + | + |
| 24 | Enterobactin | *fes* | Enterobactin esterase | + | + | + |
| 25 | Heme Uptake | *chuS* | Heme oxygenase | + | + | + |
| 26 | Heme Uptake | *chuU* | Heme permease | + | + | + |
| 27 | Heme Uptake | *chuV* | ATP-binding protein | + | + | + |
| 28 | Heme Uptake | *chuW* | Heme oxidase | + | + | + |
| 29 | Heme Uptake | *chuY* | Heme uptake protein | + | + | + |
| 30 | Heme Uptake | *shuA* | Hemoglobin receptor | + | + | + |
| 31 | Heme Uptake | *shuT* | Periplasmic binding protein | + | + | + |
| 32 | Heme Uptake | *shuX* | SHU locus protein | + | + | + |
| **Fimbrial Adhesins** | | | | | | |
| 33 | Type 1 Fimbriae | *fimA* | Fimbrial protein A chain | + | + | + |
| 34 | Type 1 Fimbriae | *fimB* | Regulatory protein | + | + | + |
| 35 | Type 1 Fimbriae | *fimC* | Chaperone protein | + | + | + |
| 36 | Type 1 Fimbriae | *fimD* | Outer membrane usher protein | + | + | + |
| 37 | Type 1 Fimbriae | *fimE* | Regulatory protein | + | + | + |
| 38 | Type 1 Fimbriae | *fimF* | Minor pilin subunit | + | + | + |
| 39 | Type 1 Fimbriae | *fimG* | Minor pilin subunit | + | + | + |
| 40 | Type 1 Fimbriae | *fimH* | Adhesin (tip protein) | + | + | + |
| 41 | Type 1 Fimbriae | *fimI* | Fimbrin-like protein | + | + | + |
| 42 | P Fimbriae | *papB* | Regulatory protein | + | + | + |
| 43 | P Fimbriae | *papC* | Outer membrane usher | + | + | + |
| 44 | P Fimbriae | *papD* | Chaperone protein | + | + | + |
| 45 | P Fimbriae | *papF* | Minor pilin subunit | + | + | + |
| 46 | P Fimbriae | *papG* | Tip adhesin | + | + | + |
| 47 | P Fimbriae | *papH* | Termination subunit | + | + | + |
| 48 | P Fimbriae | *papI* | Regulatory protein | + | + | + |
| 49 | P Fimbriae | *papJ* | Pilus assembly protein | + | + | + |
| 50 | P Fimbriae | *papK* | Minor pilin subunit | + | + | + |
| 51 | P Fimbriae | *papX* | Flagellar repressor | + | + | + |
| 52 | Common Pilus | *ecpA* | Structural subunit | + | + | + |
| 53 | Common Pilus | *ecpB* | Chaperone | + | + | + |
| 54 | Common Pilus | *ecpC* | Usher protein | + | + | + |
| 55 | Common Pilus | *ecpD* | Tip adhesin | + | + | + |
| 56 | Common Pilus | *ecpE* | Chaperone | + | + | + |
| 57 | Common Pilus | *ecpR* | Regulator | + | + | + |
| **Capsule Biosynthesis** | | | | | | |
| 58 | K1 Capsule | *kpsD* | Outer membrane protein | + | + | + |
| 59 | K1 Capsule | *kpsM* | Polysaccharide export protein | + | + | + |
| **Type III Secretion System (LEE-encoded)** | | | | | | |
| 60 | T3SS Effectors | *espL1* | T3SS effector | + | + | + |
| 61 | T3SS Effectors | *espL4* | T3SS effector | + | + | + |
| 62 | T3SS Effectors | *espX1* | T3SS effector | - | + | + |
| 63 | T3SS Effectors | *espX4* | T3SS effector | - | + | - |
| 64 | T3SS Effectors | *espX5* | T3SS effector | + | + | + |
| 65 | T3SS Effectors | *espY1* | T3SS effector | + | + | + |
| 66 | T3SS Effectors | *espY3* | T3SS effector | + | + | - |
| 67 | T3SS Effectors | *espY4* | T3SS effector | - | + | - |
| **Other Adhesins and Virulence-Associated Factors** | | | | | | |
| 68 | Adhesins | *fdeC* | Intimin-like adhesin | + | + | + |
| 69 | Adhesins | *aslA* | Putative arylsulfatase | + | + | + |
| 70 | Surface Proteins | *ompA* | Outer membrane protein A | + | + | + |
| **Stress Response and Biofilm Formation** | | | | | | |
| 71 | Curli Fibers | *csgB* | Curlin nucleator protein | + | + | + |
| 72 | Curli Fibers | *csgD* | Curlin regulator | + | + | + |
| 73 | Curli Fibers | *csgF* | Curli assembly protein | + | + | + |
| 74 | Curli Fibers | *csgG* | Curli assembly protein | + | + | + |
| **Type II Secretion System** | | | | | | |
| 75 | T2SS | *gspL* | General secretion pathway protein L | + | + | + |
| 76 | T2SS | *gspM* | General secretion pathway protein M | + | + | + |

**Abbreviations:** + indicates gene presence; − indicates gene absence. T3SS, Type III secretion system; T2SS, Type II secretion system; LEE, locus of enterocyte effacement.

**Table S4.** Metadata of the isolates and their IncFIB-IncFII plasmids used for the genomic comparisons to p10044824_1 in **Figure 1**. The three *E. coli* ST405 genomes retrieved in NCBI (SRR15116343, SRR22016559, SRR21986767) differing by only 0-2 single nucleotide polymorphisms to the three Hungarian isolates described in this study were not used in the BRIG comparisons due to the lack of complete plasmids.

| **Strain** | **Strain accession number** | **Strain origin of isolation** | **Country of isolation** | **Strain MLST (ST)** | **IncFIB-IncFII plasmid** | **IncFIB-IncFII accession number** | **Reference** |
| --- | --- | --- | --- | --- | --- | --- | --- |
| *E. coli* AR_452 | CP030331.1 | human | USA | ST156 | unnamed1 | CP030329.1 | unpublished |
| *E. coli* KY1497 | AP019803.1 | human urine | Japan | ST405 | pKY1497_1 | AP019804.1 | (41) |
| *E. coli* FUJ80154 | AP024687.1 | human | Japan | ST648 | pFUJ80154-1 | AP024688.1 | unpublished |
| *E. coli* Survcare253 | CP076305.1 | human urine | Germany | ST2851 | pS253-NDM5 | CP076306.1 | (42) |
| *E. coli* JJ1887 | CP014316.1 | human | USA | ST131 | pJJ1887-5 | CP014320.1 | (43) |
| *E. coli* ARL09/232 | NZ_CP049967.1 | human urine | New Zealand | ST7505 | pCO_Eco4457-3 | CP049970.1 | (44) |

**Table S5.** ENA accession numbers of the raw reads (Illumina and Nanopore) and hybrid assemblies of the three Hungarian *bla*_NDM-5_ carrying *E. coli* isolates.

| **WGS sequences** | **Strain name (chromosome or plasmid)^1^** | **ENA Accession No.** |
| --- | --- | --- |
| **Short-read raw sequence data (NextSeq, Illumina)** | *Escherichia coli* M2021_10044802_2_E | ERR12370011 |
|  | *Escherichia coli* M2021_10044824_1_E | ERR12370012 |
|  | *Escherichia coli* M2021_10043982_E | ERR12370013 |
| **Long-read raw sequence data (GridION, Nanopore)** | *Escherichia coli* M2021_10044802_2_E | ERR13107190 |
|  | *Escherichia coli* M2021_10044824_1_E | ERR13108142 |
|  | *Escherichia coli* M2021_10043982_E | ERR13108588 |
| **Hybrid assemblies** | *Escherichia coli* M2021_10044824_1_E (chromosome) | CP163448 |
|  | *Escherichia coli* M2021_10044824_1_E p10044824_1  (plasmid IncFIB-IncFII) | CP163449 |
|  | *Escherichia coli* M2021_10044824_1_E p10044824_2  (plasmid p0111) | CP163450 |
|  | *Escherichia coli* M2021_10044802_2_E (chromosome) |  |
|  | *Escherichia coli* M2021_10044802_2_E p10044802_1  (plasmid IncFIB-IncFII) | CP163452 |
|  | *Escherichia coli* M2021_10044802_2_E p10044802_2  (plasmid IncX4) | CP163453 |
|  | *Escherichia coli* M2021_10044802_2_E p10044802_3  (plasmid p0111) | CP163454 |
|  | *Escherichia coli* M2021_10043982_E p10043982_1  (plasmid IncFIB-IncFII) | CP163455 |
|  | *Escherichia coli* M2021_10043982_E p10043982_2 (plasmid IncX4) | CP163456 |
|  | *Escherichia coli* M2021_10043982_E p10043982_3 (plasmid p0111) | CP163457 |

^1^ The chromosomal assembly of isolate M2021_10043982_E could not be completed due to technical difficulties during total genomic DNA extraction for long-read sequencing; only plasmid assemblies are deposited for this isolate.
