# Supplementary material for: Human-associated NDM-5-producing multidrug-resistant Escherichia coli detected in retail beef and pork in Hungary, 2021
Source: Front Bioinform. 2026 Mar 24;6:1793862. doi: 10.3389/fbinf.2026.1793862 (PMC13053247; doi:10.3389/fbinf.2026.1793862)

Plots showing read length distribution vs. read quality **before** trimming with Filtlong

Plots showing read length distribution vs. read quality **after** trimming with Filtlong

E. coli **strain M2021_10043982_E**

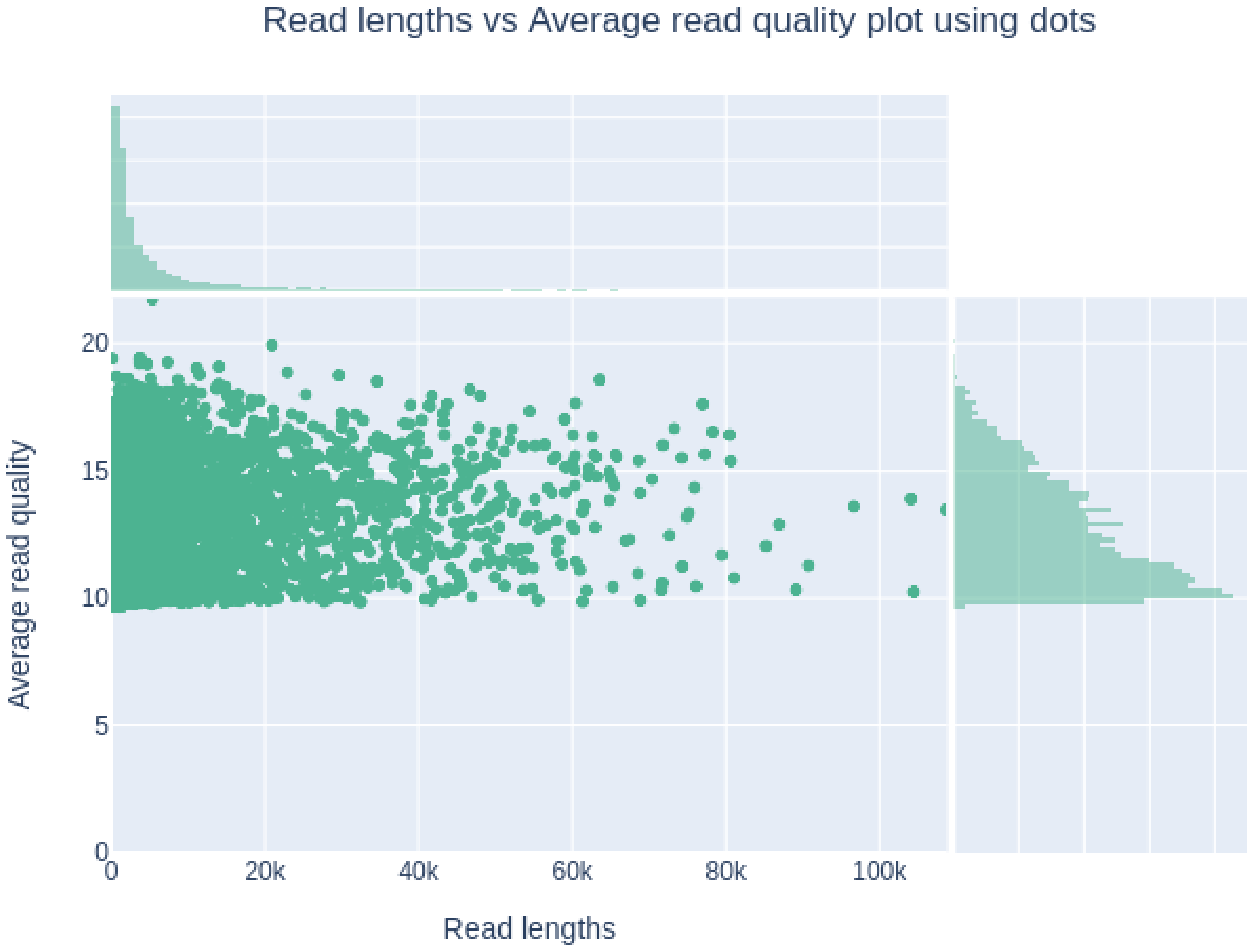

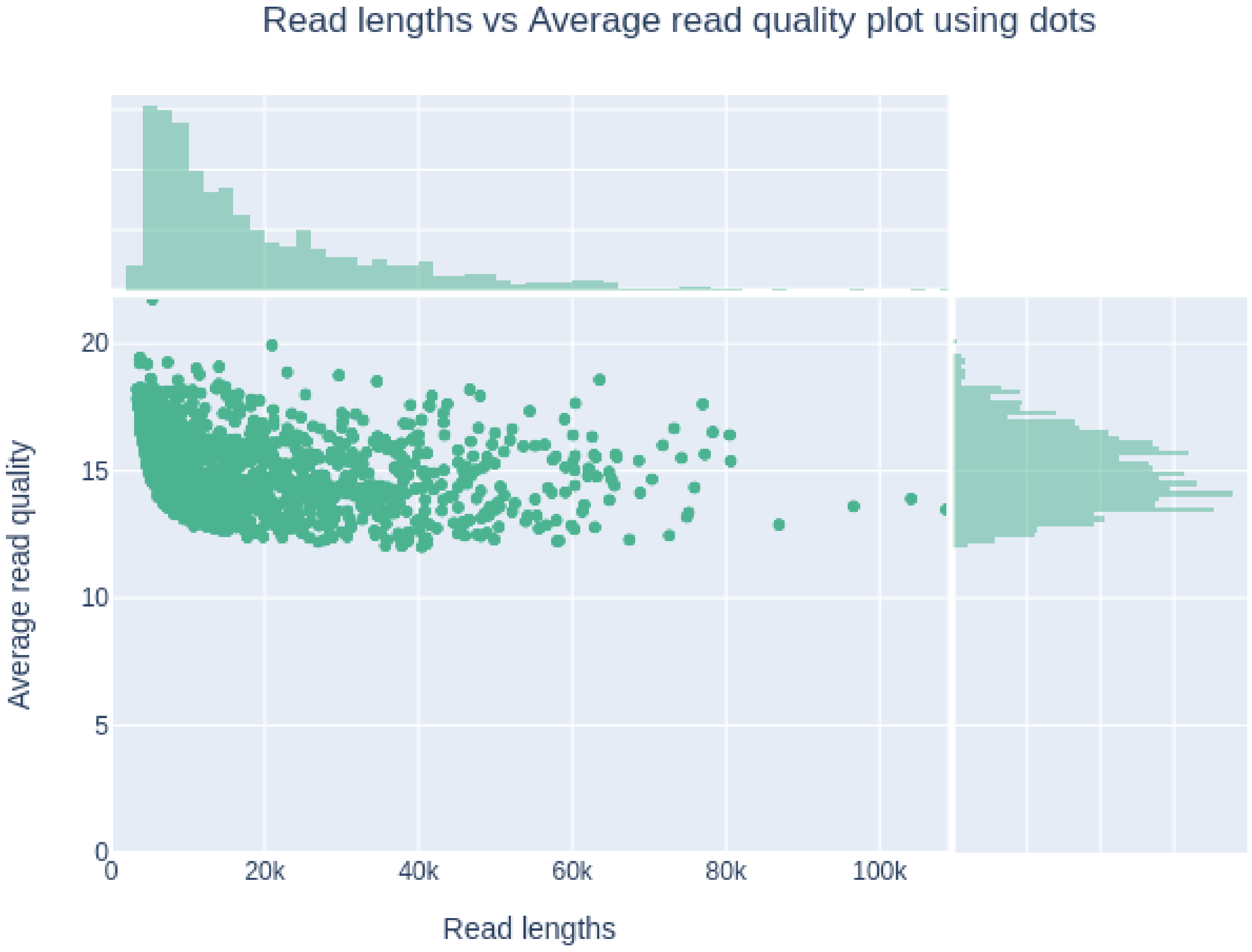

E. coli **strain M2021_10044802_2_E**

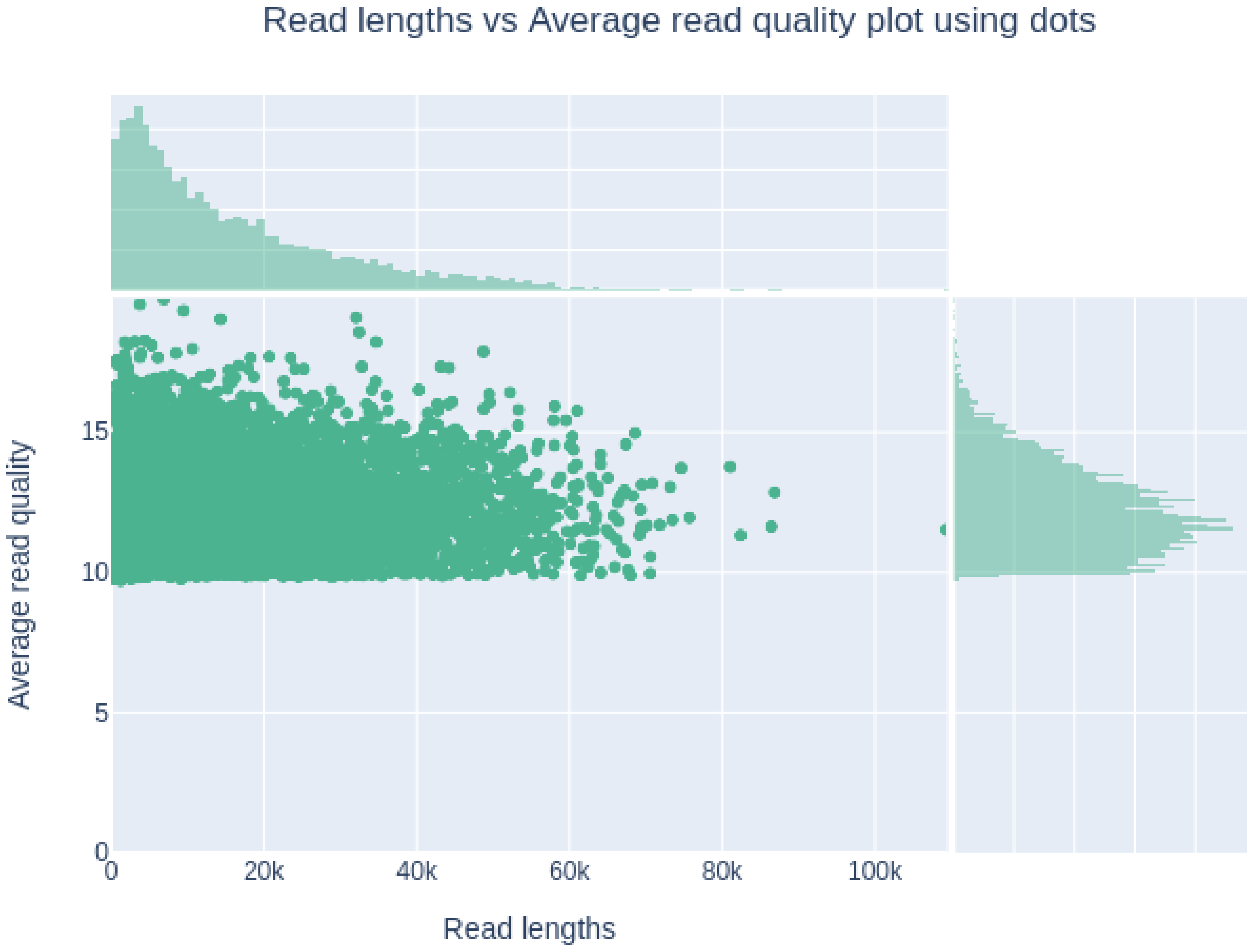

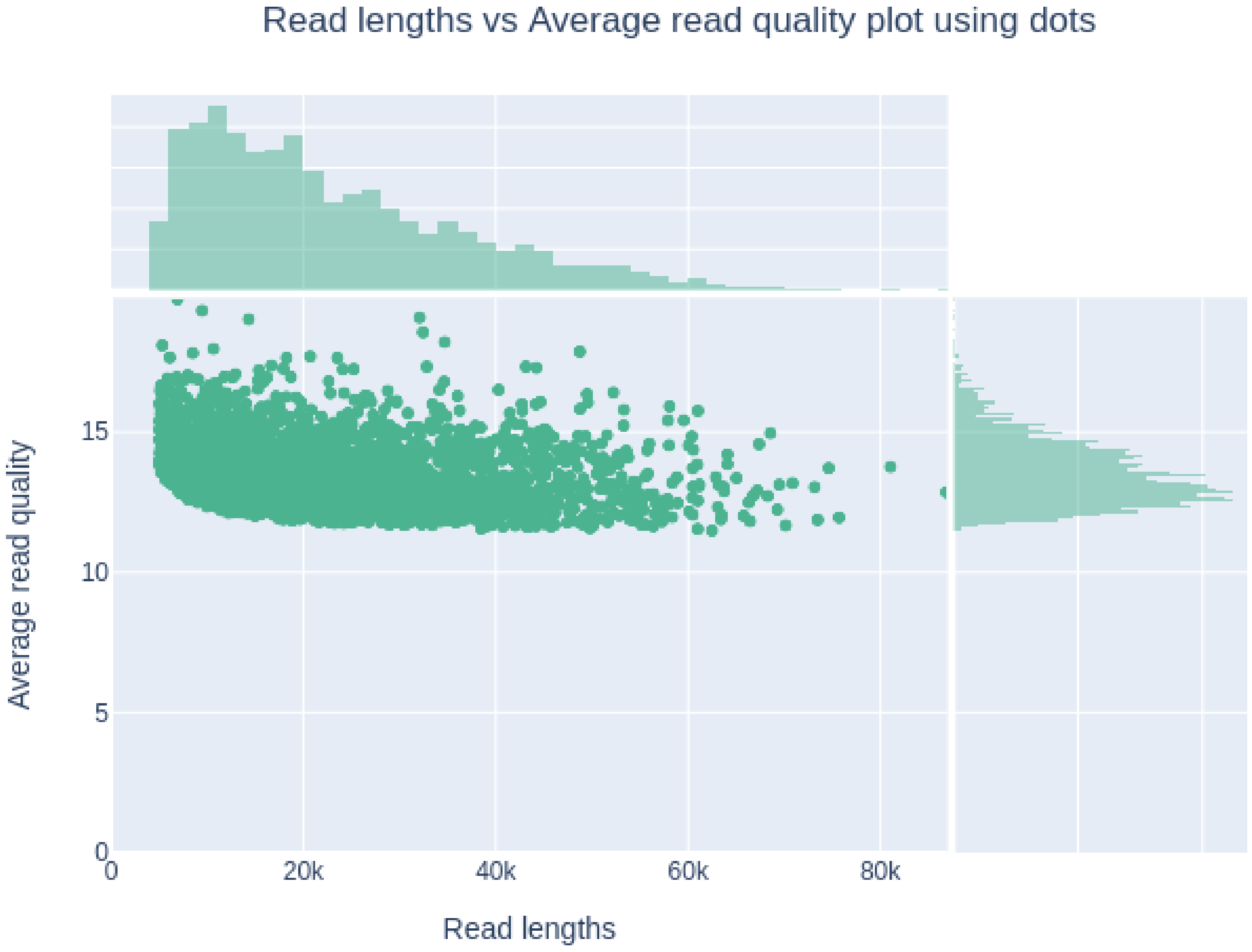

E. coli **strain M2021_10044824_1_E**

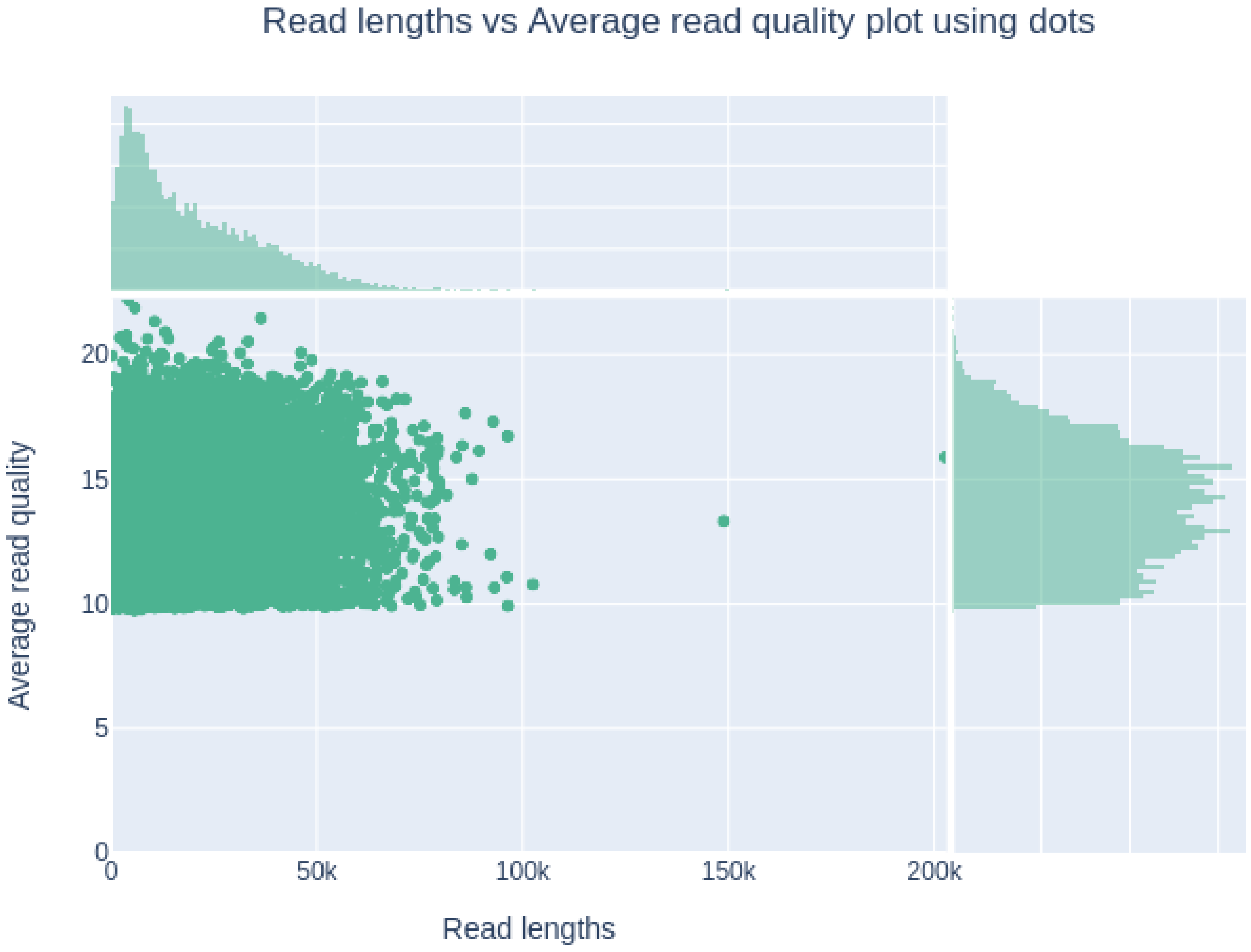

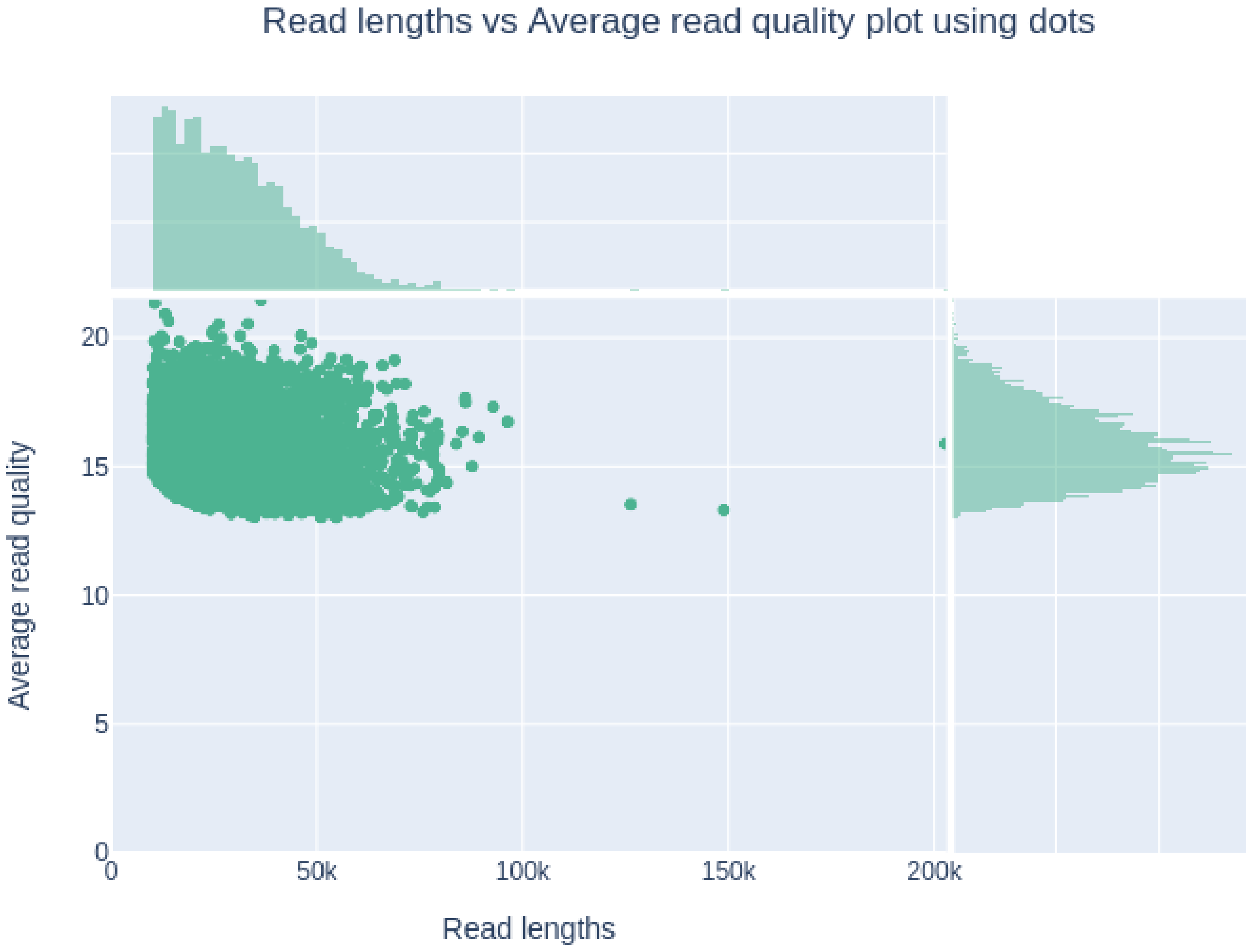

Supplement: Supplementary file 2 [file DataSheet1.pdf]
